# Supplementary material for: Multi-laboratory assessment of reproducibility, qualitative and quantitative performance of SWATH-mass spectrometry
Source: Nat Commun. 2017 Aug 21;8:291. doi: 10.1038/s41467-017-00249-5 (PMC5566333; doi:10.1038/s41467-017-00249-5)
Supplement: Supplementary file 1 — Supplementary Information [file 41467_2017_249_MOESM1_ESM.pdf]

File Name: Supplementary Information
Description: Supplementary Figures, Supplementary Notes, Supplementary Methods and Supplementary References

File Name: Peer Review File
Description:

File Name: Supplementary Dataset 1
Description: This table describes how the different peptide groups were diluted serially 3-fold into the HEK293 background to create the 5 different samples.

File Name: Supplementary Dataset 2
Description: List of heavy labeled synthetic peptides and the group they were mixed into.

File Name: Supplementary Dataset 3
Description: OpenSWATH/pyprophet-cli results including protein and peptide peak groups detected for results aggregated across all files in the dataset

File Name: Supplementary Dataset 4
Description: Peptide Peak areas (Sum of 4 Fragment Ions) for SIS peptides from sample S4

File Name: Supplementary Dataset 5
Description: CVs determined from SIS peptide peak areas (sum of 4 fragment ions) from sample S4

File Name: Supplementary Dataset 6
Description: Normalized protein abundances for all SWATH files in the study (229) estimated by summing the peak areas the top 5 fragment ions from the top 3 peptide peak groups (see methods for details)

File Name: Supplementary Dataset 7
Description: Protein abundances from HEK293 matrix with 80 % completeness

File Name: Supplementary Dataset 8
Description: Responses from SIS peptides in dilution series

File Name: Supplementary Dataset 9
Description: Averaged response from SIS peptides in dilution series

File Name: Supplementary Dataset 10
Description: % detection of SIS peptides for SWATH and MS1

File Name: Supplementary Dataset 11
Description: Responses from SIS peptides in dilution series - MS1 C12

File Name: Supplementary Dataset 12
Description: Averaged response from SIS peptides in dilution series from MS1 XICs

File Name: Supplementary Dataset 13

Description: Nanoflow LC-MS configurations for all sites

File Name: Supplementary Dataset 14
Description: LC gradient information for each site

File Name: Supplementary Dataset 15
Description: 64 variable window setup for SWATH acquisition

File Name: Supplementary Dataset 16
Description: Acquisition order of samples

File Name: Supplementary Dataset 17
Description: MultiQuant™ method for quantification of SIS peptides

Supplementary information for:

Multi-laboratory assessment of reproducibility, qualitative and quantitative performance of SWATH-mass spectrometry

Supplementary Note 1 – Initial quality assessment round

In order to standardize the acquisition protocol and to generate an initial quality assessment, we first asked each participating lab to acquire 5 replicate injections of a test sample containing the HEK293 background with retention time calibration (iRT) peptides added. The data arising from the analysis of this initial phase was used to improve the data acquisition protocol and prompt any sites that were having any issues. The number of peptides detected (<1% FDR) and quantified with %CV < 20 across 5 replicates were compared with various LC/MS performance attributes (Supplementary Fig. 1).

From the initial data analysis, there were 3 sites that were showing slightly lower performance that were asked to repeat the QC round of analysis after fixing specific issues. Improvements in peptide quantification were achieved in all cases.

As an example, the initial QC data collected for Site 4 showed lower numbers of quantified peptides as well as a number of missing iRT peptides (the early eluting peptides). Investigation of the MS total ion chromatograms also indicated a reproducibility issue during the early portion of the gradient. This was traced back to a problem with the autosampler method and upon correction, results improved to be more consistent with other sites, from 18559 to 34394 peptides quantified. This can be observed in Supplementary Figs. 1a and 1c as an increase in average peptide area of the iRT peptides (as 4 additional peptides were measured) and a decrease in average retention time variation.

Supplementary Note 2 – Control of false discovery rate (FDR)

With increasing data set size (number of runs and number of spectra), the error-rate control methods in discovery proteomics were extended over the past decade to account for the observation that the FDR at protein level could be significantly inflated compared to PSM, or peptide level due to the tendency of true positive PSMs to distribute non-uniformly on target proteins whereas false positive PSMs are uniformly distributed[1]. A key feature of the data that influences the error-rate is the ratio of undetectable peptides to the total number of peptides in the search database which is referred to as π_0. In DDA based methods where MS2 spectra are typically searched against all protein sequences for a given organism the fraction of peptides

that are in the database but are not present at a detectable level in the sample is large (e.g. $\pi_0 \approx 0.8$) leading to significant inflation of the FDR when moving from PSM to protein level. Similar inflation of the error-rate can occur as the number of samples analyzed increases unless the FDR is controlled in a global manner. In SRM studies the number of targets is typically small and the prevalence of detectable targets is typically high (e.g. $\pi_0 \approx 0.05$) and, as such, this problem can be ignored.

Since the introduction of SWATH-MS the primary method for control of the FDR in peptide-centric data analysis has been based on methods originally adapted from SRM based analysis implemented in the mProphet[2] software. In this strategy a null distribution is parameterized using a set of decoy peptide queries (non-parametric methods are also available). P-values for the target peptide queries are then computed and corrected for multiple testing to derive q-values[3] allowing for control of the FDR on the peptide query-level. In SWATH-MS, or other related DIA approaches, the magnitude of π_0 depends strongly on the size of the spectral library used to generate the peptide query parameters, and more specifically the number of peptide queries. In an equivalent way to the problem discussed above in discovery proteomics, error-rate control on peptide query-level only is insufficient to infer sets of proteins in peptide-centric analysis of DIA data, and this problem scales with the magnitude of π_0.

In early SWATH-MS studies spectral libraries were typically generated using side-by-side DDA analysis of the same samples and, as such, π_0 was small (similar to the SRM case mentioned above). Thus, error rate control at the inferred protein level was not critically important. In later studies we tested a repository-scale spectral library containing peptide query parameters for 10,000+ proteins[4] to generate peptide queries and employed protein level FDR control in single SWATH-MS runs using the MAYU software[1] to account for the increase in π_0. In the inter-lab SWATH-MS study described here we chose to use the same repository scale spectral library to simulate an exploratory analysis where sample-specific spectral libraries might not be available and to demonstrate the feasibility and potential of using such large-scale libraries. The result was a $\pi_0 \approx 0.6$ and given that we were collectively analyzing 200+ SWATH-MS runs in combination with such a large-scale spectral library we took particular precautions for error-rate control. A full and general examination of issues relating to the error-rate control in DIA data analysis, including a detailed description of the methods used here, is provided in the accompanying perspective manuscript[5]. This includes the extension of FDR estimation from peptide query level to the peptide, and inferred protein levels. This also includes computing q-values for every peptide query on a sample-by-sample basis which provides a matrix-like representation where the question whether a particular peak group or inferred protein was detected or not in a given sample is answered (referred to as experiment-wide context in the accompanying perspective[5]); or, computing q-values based on the best scoring instance of a

given peptide query over the whole dataset where we define a global list of peak groups or inferred proteins detectable in the entire study (referred to as global context). In this study, the false discovery rate (FDR) was controlled at 1% at the peptide query, and protein levels using the q-value approach in the global context, and at 1% peptide query FDR on a sample-by-sample basis (experiment-wide context). In this study we have chosen to include proteins represented by only one proteotypic peptide, however, as we have applied protein level FDR control we expect that the false discoveries should still be within or approximated by the thresholds chosen[1].

The difference between the sets of inferred proteins reported when the dataset was analyzed in an aggregated manner where all 229 SWATH-MS acquisition were collectively analyzed, or site-by-site where 11 separate analyzes were performed, as shown in **Figures 2a and 2c** respectively, can be explained by this filtering strategy. In the first instance the global context described above refers to all SWATH-MS runs from all sites, and in the second instance the global context refers to only 21 files analyzed at a given site. That is, in our analysis a protein is inferred in a given sample if a peak group mapping to that protein is detected at the 1% peptide query FDR threshold as long as the peptide has been detected elsewhere in the experiment with a score passing the 1% protein FDR threshold (i.e. global context).

Supplementary Note 3 – Comparison between MultiQuant and OpenSWATH data analyses

MultiQuant software was used for the concentration curve analysis because linearity of fit, %CV at each point and accuracy of each fit are easily evaluated rigourously using established methods for targeted analysis. However, at the scale of the HEK293 data analysis inspection of all data points is not possible. Software tools such as OpenSWATH have been developed for large scale integration and FDR analysis. We have previously analysed the correspondance of a fully automated OpenSWATH analysis to a manually curated analysis of a gold standard data set and found that the they are in good agreement within the boundaries of the analysis and accounting for the FDR threshold[6]. Because the SIS peptides were analysed both by MultiQuant (including inspection) and OpenSWATH (fully automated) we can also compare the results in this data set.

Supplementary Figure 18 shows a comparison of the lowest concentration at which the SIS peptide can be quantified/detected. The threshold is LLOQ in the case of the MultiQuant analysis (using S/N, %CV, accuracy thresholds as described) and the threshold is FDR based for OpenSWATH. The analysis of the peptides in groups A and B which had concentrations spanning the detection limit (mid attomole – low femtomole range on column) showed that there is a good correspondence between the LLOQ (MultiQuant) and the lowest concentration detected using FDR-based analysis (OpenSWATH). That is, of the 8 detectable peptides in groups A and B, for 6

of these the LLOQ was at the same concentration as the lowest detectable by the FDR-based method, and for the remaining 2 peptides there was a difference of one 3x dilution step (one of these peptides was lower in MultiQuant analysis and one in OpenSWATH).

Supplementary Figure 19 shows the full dilution curves as determined by MultiQuant or by OpenSWATH analysis. As expected, the analysis of SIS peptides using Multiquant, including inspection and peak re-integration where required, is superior to the fully automated analysis. In particular, in the highest concentration range (3-10 picomole on column) OpenSWATH underestimates the peak areas for most peptides significantly and fails to maintain linearity at the top end of the dilution curve. This is a known issue in OpenSWATH for peptides in the picomole range where the chromatographic peak width increases significantly (peak widths of 2-3 minutes sometimes with multiple maxima/minima). To determine if this would significantly affect the quantitative performance in OpenSWATH analysis of the HEK293 proteome, we plotted the peak area distribution of these peptides on the same scale next to the dilution curves. In this sample we do not have any endogenous peptides that extend into the highest concentration range which is problematic for OpenSWATH peak integration. In a previous study of bacterial proteomics we did see this peak integration issue for some very high abundance stress response proteins and we solved this by manually integrating these atypical peaks in a different software[7]. This was feasible with moderate effort because the number of peptides where such saturation issues were detected was small. Ultimately the flexibility of the peak integration in OpenSWATH should be improved to deal with sample types that contain peptides in the picomole on column range, however, this is outside the scope of the current study.

Supplementary Note 4 – Assessment of MS1 vs. SWATH-MS data

In each SWATH-MS acquisition data file, both MS1 and MS2 data were collected in every cycle. This allowed for the comparison of quantitative parameters between the two acquisition modes, presented here for the 30 stable isotope standard peptides diluted in the HEK293 matrix. For MS1 data, high resolution extracted ion chromatograms (XICs) of the C12 and first C13 isotope were generated using 0.02 m/z XIC width in MultiQuant Software 3.0. The SWATH-MS data was also extracted in MultiQuant using the top 4 fragment ions per peptide (C12 ion), using 0.05 m/z XIC widths. As shown in Figure 5c, it was observed that the average % detection curves were typically lower in the SWATH-MS data (the MS2 level data) than the MS1 data. Most often, this was because the interferences observed in the SWATH-MS XICs were less than in the MS1 data. Using the MS2 data for quantification provided an added level of specificity for the measurement of the peptides in a very complex matrix. Several examples are shown in Supplementary Fig. 19. In addition, the average Signal to /Noise (S/N) values were recorded for each peak at each

concentration, and the S/N values were averaged per concentration (Supplementary Fig. 20). The average SWATH-MS S/N curve was higher than the S/N curve for the MS1 data, again supporting that the main source of improved detection in the SWATH-MS data was because of the removal of interferences and noise (Supplementary Fig. 21). The S/N was reported in MultiQuant Software for the peptide at each concentration using the relative noise approach.

This % detection data in Figure 5b was generated using the XICs from the C12 ion to construct the SWATH-MS concentration curves for each of the dynamic range peptides. In other work, multiple precursor isotopes have been used for MS1 quantification so this was also investigated here. In general, adding the second isotope to the quantification curves had minimal impact on the detection limits (Fig. 5c) and the average S/N observed was found to decrease slightly when summing in the second isotope (Supplementary Fig. 20). In assessing the MS1 data, it was shown that the resultant S/N value was variable and it was not clear as to whether the interference was introduced from the C12 isotope or by including the first C13 isotope.

Supplementary Note 5 – Discussion on data completeness/missing data

The problem of missing data in a peptide or protein vs sample data matrix is a general problem for quantitative proteomics and high dimensional molecular profiling datasets broadly speaking. One of the primary goals of SWATH-MS and related DIA approaches is to reduce the number of missing values in quantitative proteomics datasets for technical reasons, however, this is not completely effective for a variety of reasons. Some of these are technical or stochastic (i.e. a sample specific interference precluded detection of the target; targets close to the noise level may, for stochastic reasons, be above or below the score threshold; pre-analytical variation such as in sample preparation, etc). Alignment strategies, such as TRIC which we cite in the main text but did not apply for transparency and simplicity reasons mentioned in the text, can improve this in some cases. The technical reasons for missing data, arising primarily from instrument variation or stochastic effects, is the type that is reported in our study (in our study sample preparation variation is not considered but could come in this category if it were assessed).

However, there are also biological reasons for missing data and these were not assessed in our study (i.e. the protein is not present in a given sample or rather that the abundance falls below the detectable limit in a given experimental condition). In real experiments in which biologically different samples are compared it is challenging to confidently distinguish biological and technical reasons for missing data because there is no ground truth. A simulated ground truth in which biological reasons for missing data are calculated could serve as a useful approximation to study the problem. Here, we consider the 'LFQbench' study[8] as such an approximation. In this study the authors used proteomes from three different species that were mixed in different

ratios and quantified. Here, biological reasons for missing data is essentially simulated because some proteins in the low abundance range will be detectable in one condition but not in the second condition. The authors demonstrate this to some extent in that they observed this type of condition specific missingness more often when the expected ratio of the proteins from a given species was increased from 1:2 to 1:4 to 1:10 (see Supp. Fig. 17 of this paper – here this is referred to as incomplete cases). We suggest that from this data an idea of how biological roots of missing data affects data completeness can be established and we further suggest a future study that combined our inter-laboratory design with this type of multi-species proteome sample with known mixing ratios could potentially decompose the differences between technical/biological missingness.

However, it is also important to state that there is no way to solve this biological missingness problem from a data acquisition or signal processing perspective. The only way to ultimately deal with this is via re-quantification type approaches where an integrated noise value is included in the data matrix to act as upper boundary for the purpose of downstream analysis[9], or values are imputed into the data matrix using statistical methods[10]. The utility of these methods are under investigation by many groups but we suggest that this is an open research question that requires further work. A common heuristic to manage this problem is to use a completeness filter to make the data more suitable for statistical analysis perspective. In this study we used a cutoff value of 80% completeness for downstream analysis, however, this threshold is somewhat arbitrary and would probably need to be reduced in samples that have many experimental groups/conditions to account for true biological missingness in several experimental conditions.

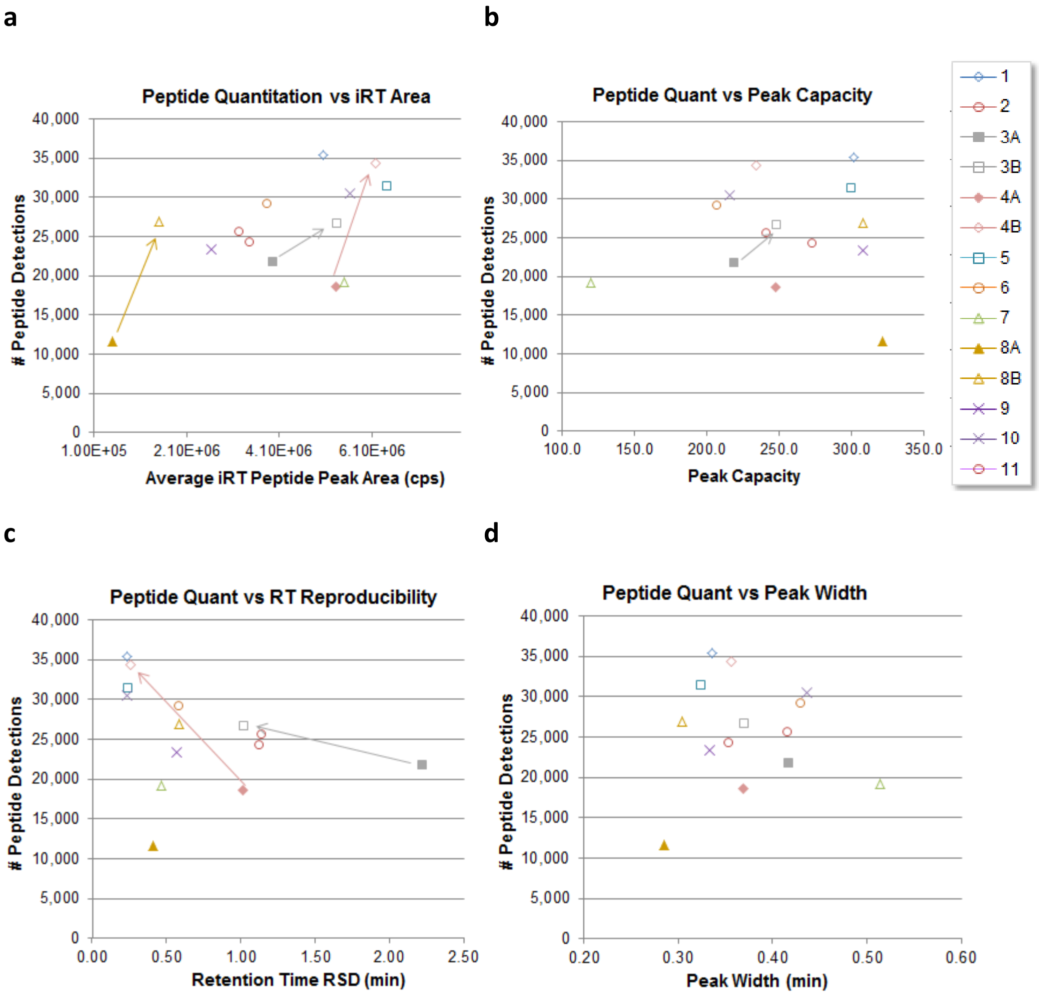

Supplementary Figure 1 – Plots of various performance attributes vs the number of peptides quantified (a) The peak areas for all the retention time calibration (iRT) peptides were averaged and this value was compared to the number of peptides quantified. This metric provided the best prediction of peptides quantified. The arrows and A/B nomenclature indicate the 3 sites that were asked to repeat the quality assessment round to improve results. The arrows are included to indicate significant changes in performance after the initial quality check (b) Peak capacity is the total retention time spread of the iRT peptides divided by the average chromatographic peak width. There wasn't a significant correlation between this metric and quantified peptides. (c) The reproducibility of retention time of the iRT peptides showed minimal correlation with peptides quantified; however larger deviations did seem to be detrimental. (d) The peak width of the iRT peptides also did not show a strong correlation with quantified peptides.

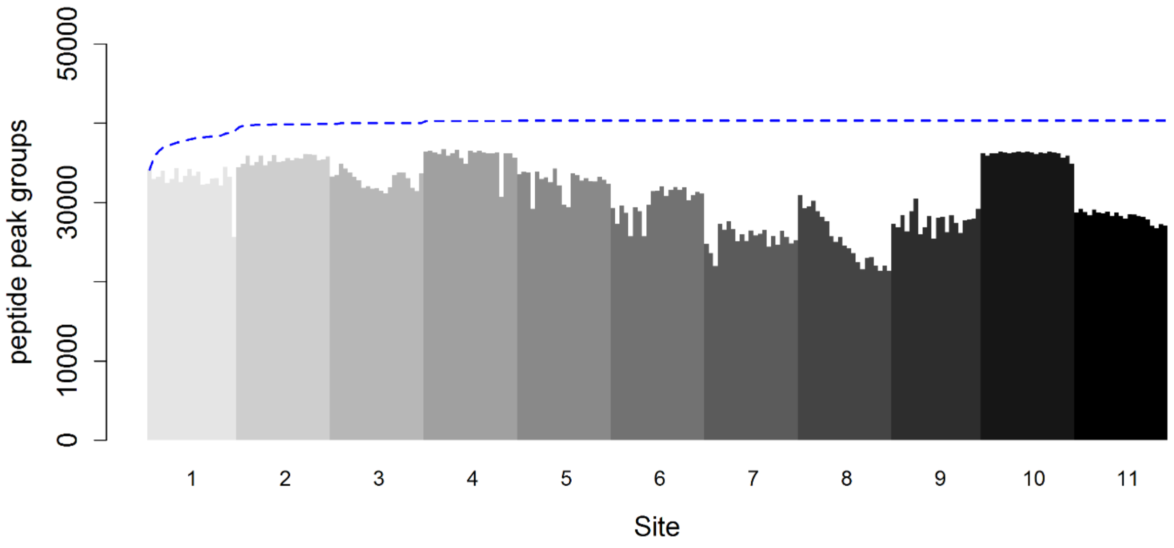

Supplementary Figure 2 – Peptide peak group detected at 1% FDR – analysis aggregated over all sites

The number of peptide peak groups detected in each of the 229 SWATH-MS analyses is shown ordered by site of data collection and then chronologically by time of acquisition. The blue line indicates the cumulate set of proteins detected with each new sample moving from left to right. This is equivalent to Figure 2a, except plotted at the peptide peak group level instead of the protein level.

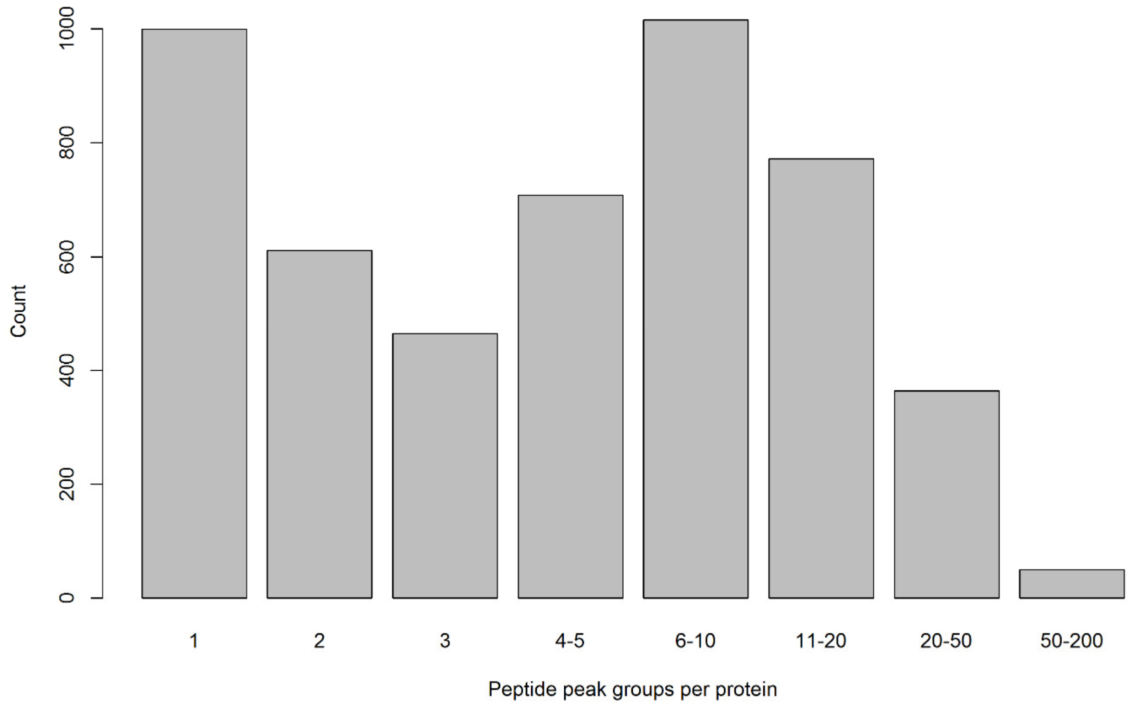

Supplementary Figure 3 – Number of peptide peak groups detected per protein

The distribution of the number of peptide peak groups detected per protein over the whole data set.

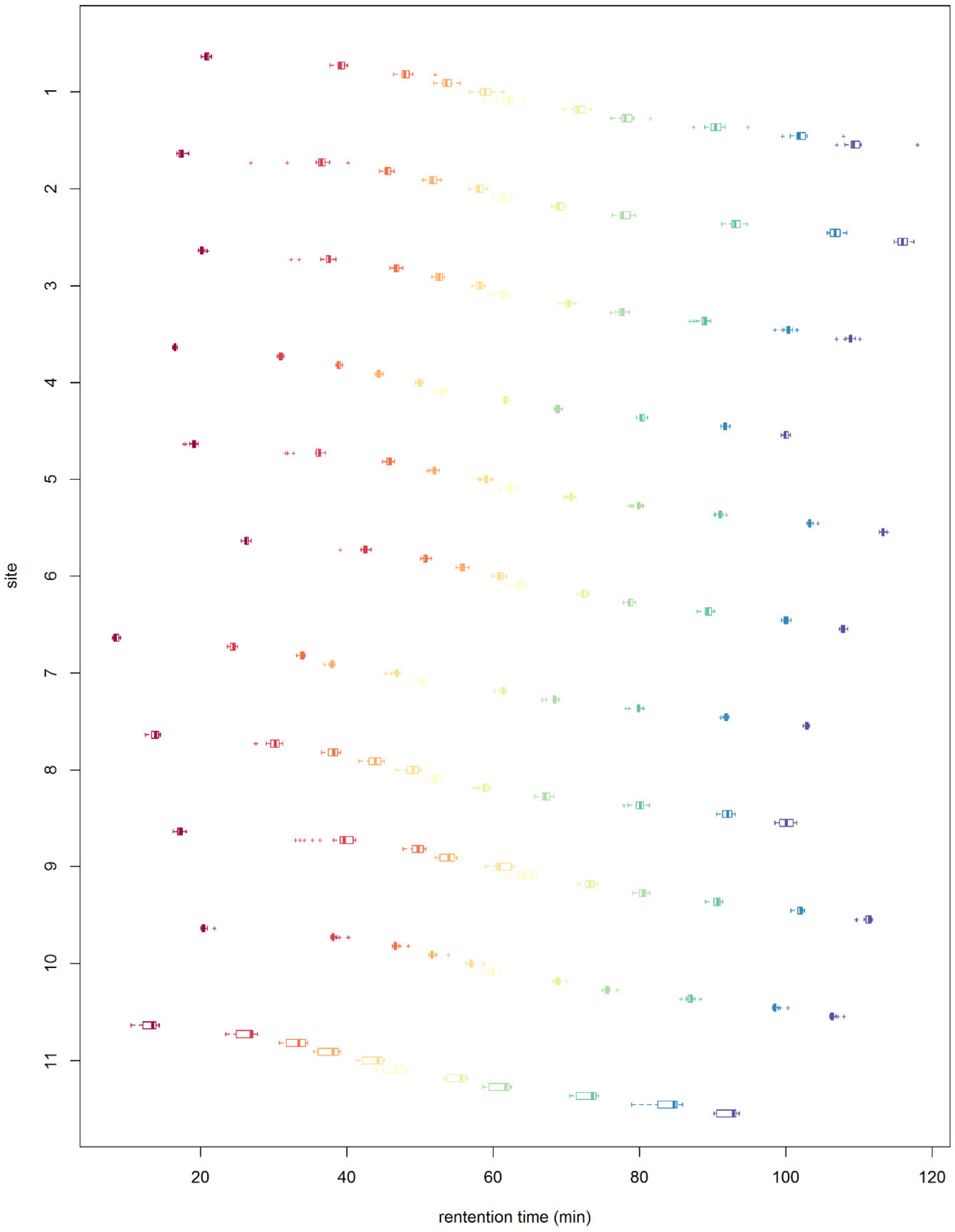

Supplementary Figure 4 – Variation in absolute retention times across sites

The variation of retention times in absolute terms across sites is visualized using the 11 iRT peptides which were spiked into the samples. The boxes represent the variation within one site across the 21 SWATH acquisitions. Boxes indicate the interquartile range of the retention time of a given peptide at a given site with the dividing line indicating the median. The maximum value of the whiskers is 1.5x the interquartile range with values outside the whiskers plotted as crosses.

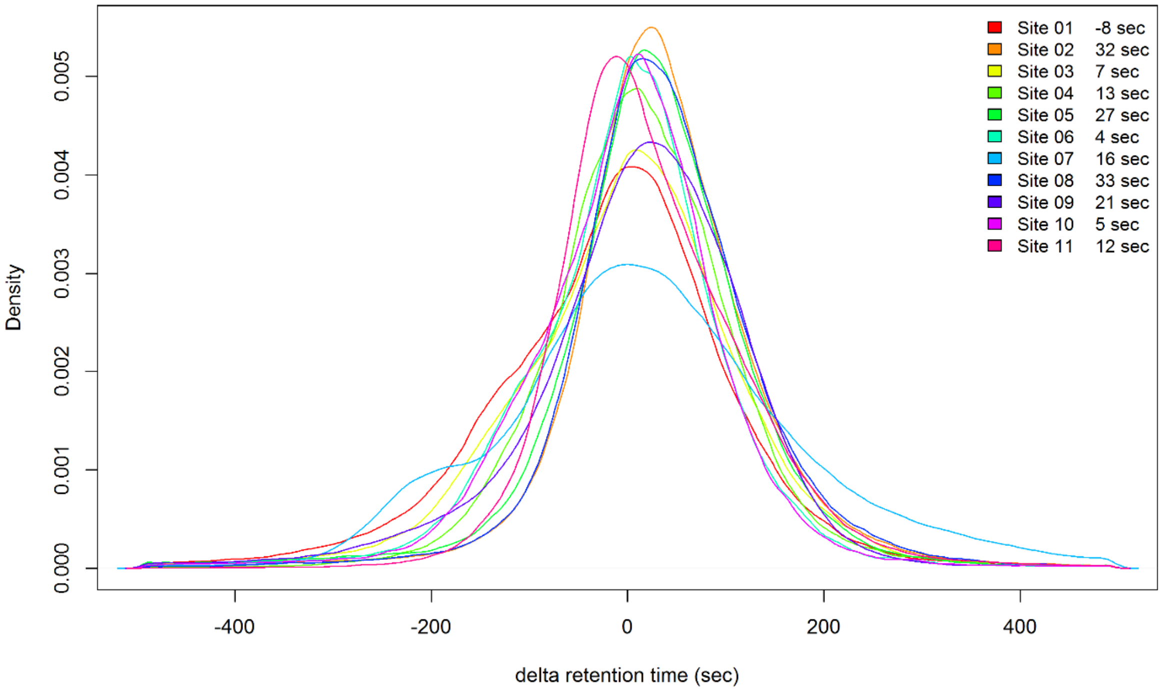

Supplementary Figure 5 – Distribution of deviations from the predicted retention times

The deviation from the predicted retention time from all peptide peak groups detected from the HEK293 proteome each site is shown in density plots. The predicted retention time is derived by projecting the retention time for a given peptide query which is stored in the normalized iRT space[11,12] on to the retention time space for each individual SWATH acquisition. In this plot we show the deviation of the measured retention time from the predicted retention time. The median for each site is shown in the legend. The distributions of deviations from the predicted retention times fall well within the extraction 15 minute window (± 7.5 min) used in the OpenSWATH analysis.

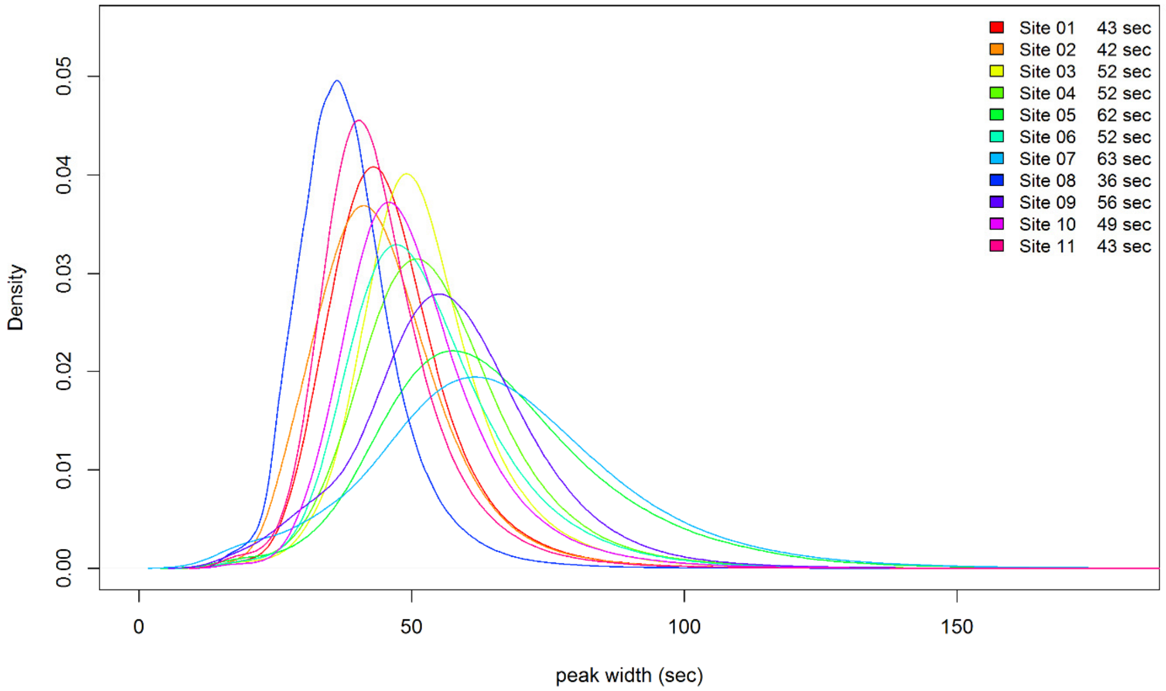

Supplementary Figure 6 – Distribution of chromatographic peaks widths

The distribution of chromatographic peak widths (at base) for peptide peak groups detected from the HEK293 proteome at each site is shown in density plots. The median for each site is shown in the legend.

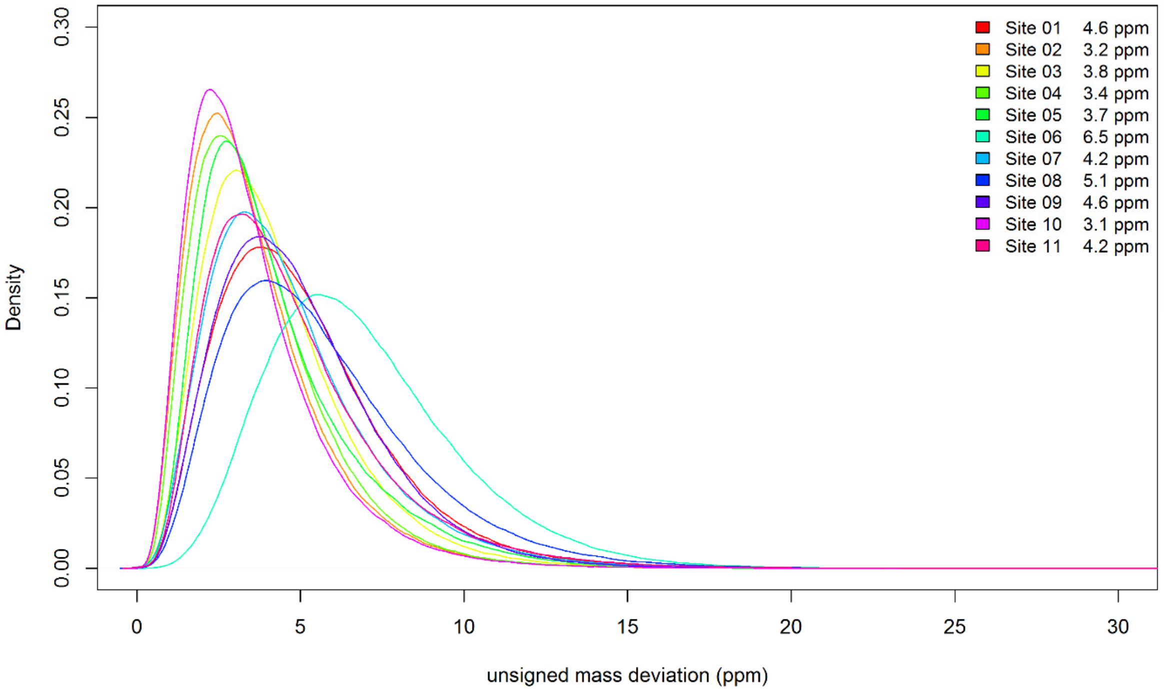

Supplementary Figure 7 – Distribution of mass errors in SWATH-MS data

The unsigned mass error in the SWATH-MS data from each site is shown in density plots. The mass error is calculated for each peptide peak group as the sum of the individual fragment ion mass deviations in ppm divided by the number fragment ions extracted. The median for each site is shown in the legend. The distributions of mass errors fall well within the extraction width of 75 ppm used in the OpenSWATH analysis.

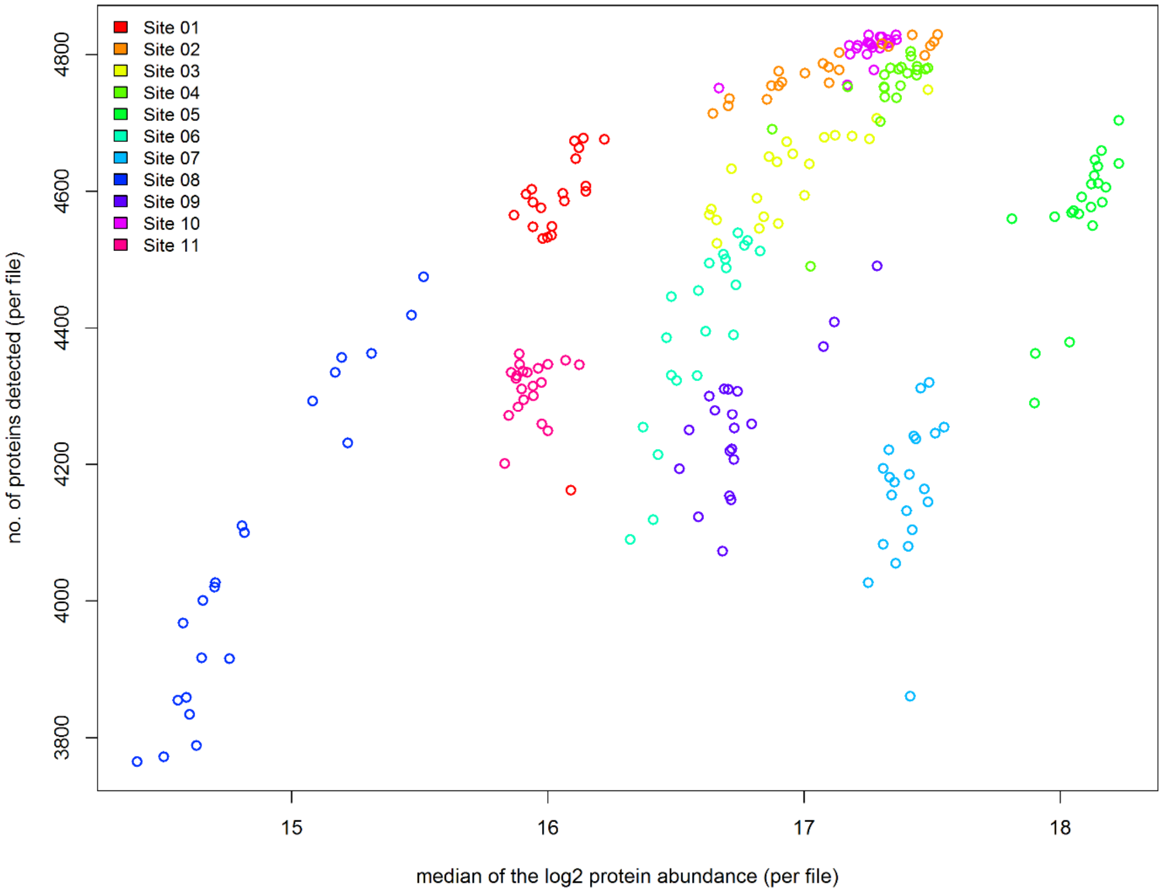

Supplementary Figure 8 – Correlation of number of proteins detected with median protein abundance

The number of proteins detected from the HEK293 proteome in each SWATH acquisition from each site is plotted against the median protein abundance from that SWATH acquisition. The number of proteins detected in a SWATH-MS acquisition at a given site was only moderately correlated with the median protein abundance in that acquisition (Pearson correlation 0.54, or 0.29 when files from site 8, which experienced some technical difficultly, are removed) indicating that the signal intensity is not a direct predictor of data quality. A similar result is shown in Supplementary Fig. 1a where the number of peak groups detected is correlated with the peak areas for iRT peptides (another measure of instrument response factor).

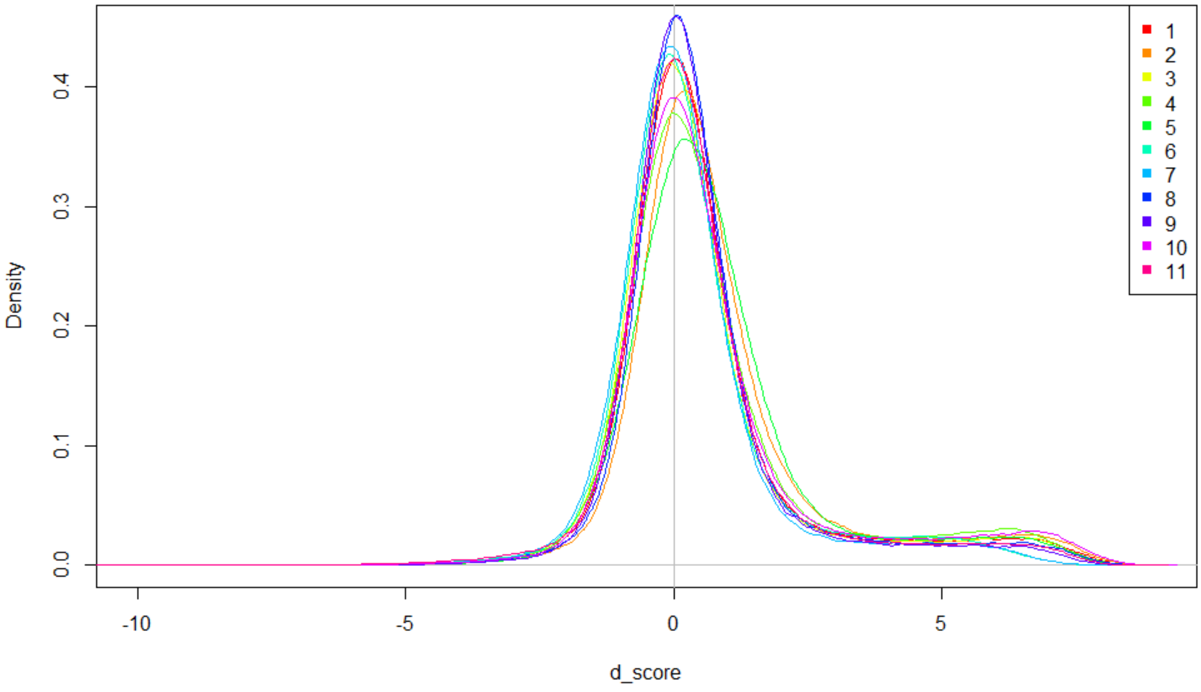

Supplementary Figure 9 – Site-wise discriminant score distributions from OpenSWATH/PyProphet analysis

The distribution of the discriminant score generated by OpenSWATH/PyProphet analysis is plotted for the best group for all target and decoy peptide queries for 1 representative files from each site of data acquisition.

a

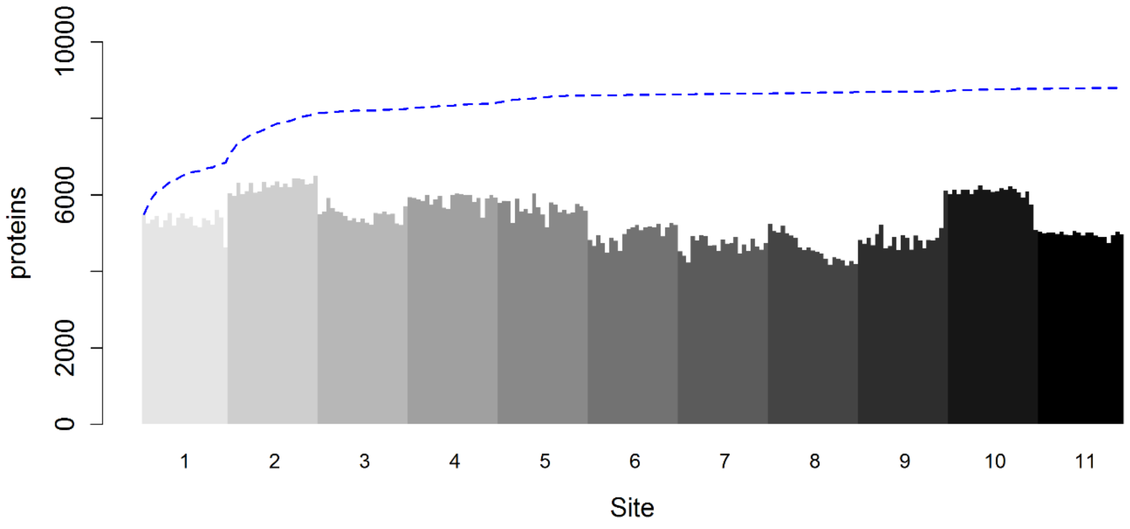

b

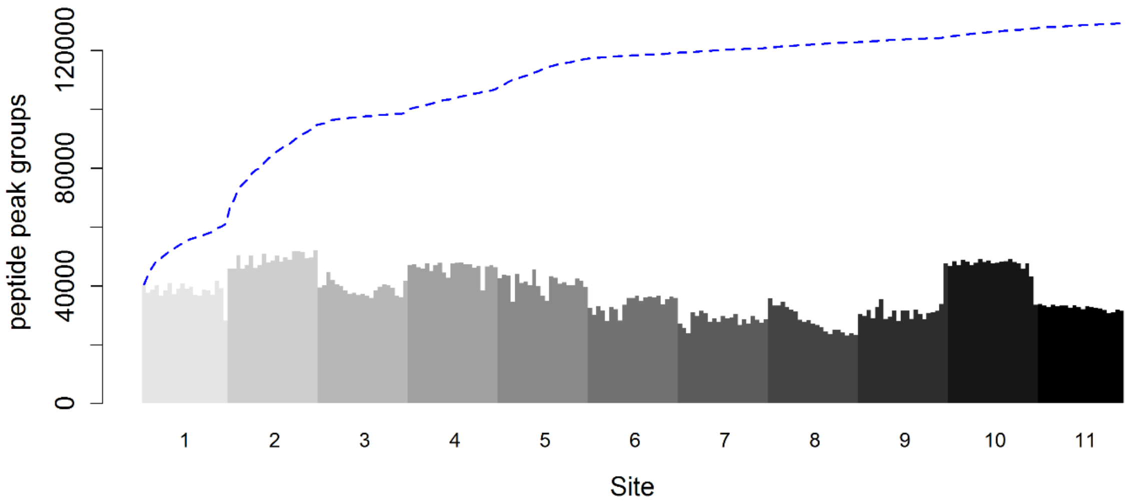

Supplementary Figure 10 – Peptide peak groups and proteins detected without global context FDR control

The accumulation of (a) proteins and (b) peptide peak groups detected when the FDR was controlled at 1 % only at the peptide query level and not protein level, and only at on a sample-by-sample basis (experiment-wide context) and not across the whole dataset (global context).

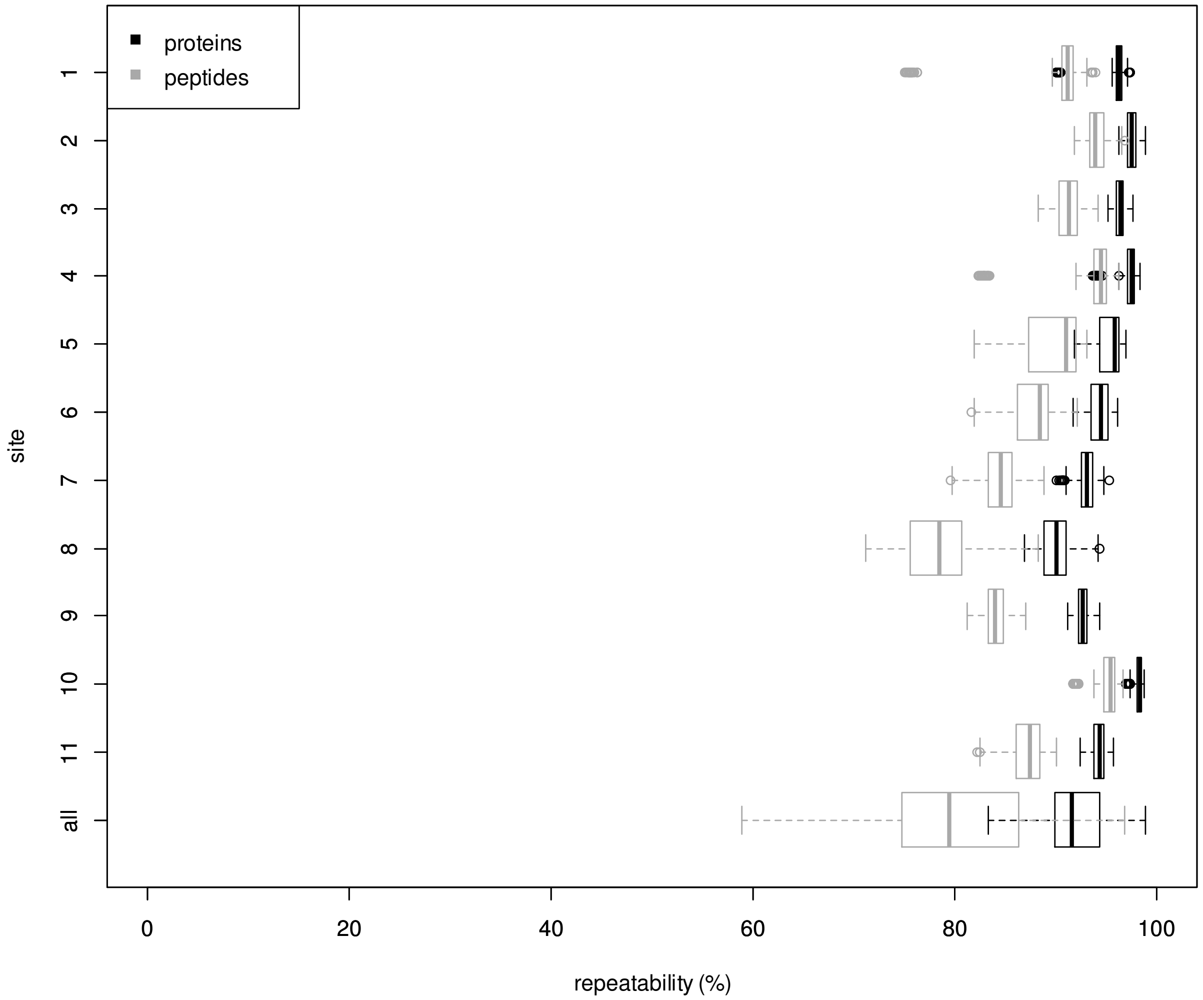

Supplementary Figure 11 – Repeatability of peptide and protein detection

The repeatability[13], defined as the intersect divided by the union between the peptide or proteins detected from 2 data files, computed pairwise within the site of data collection (1-11) or across the entire dataset (all).

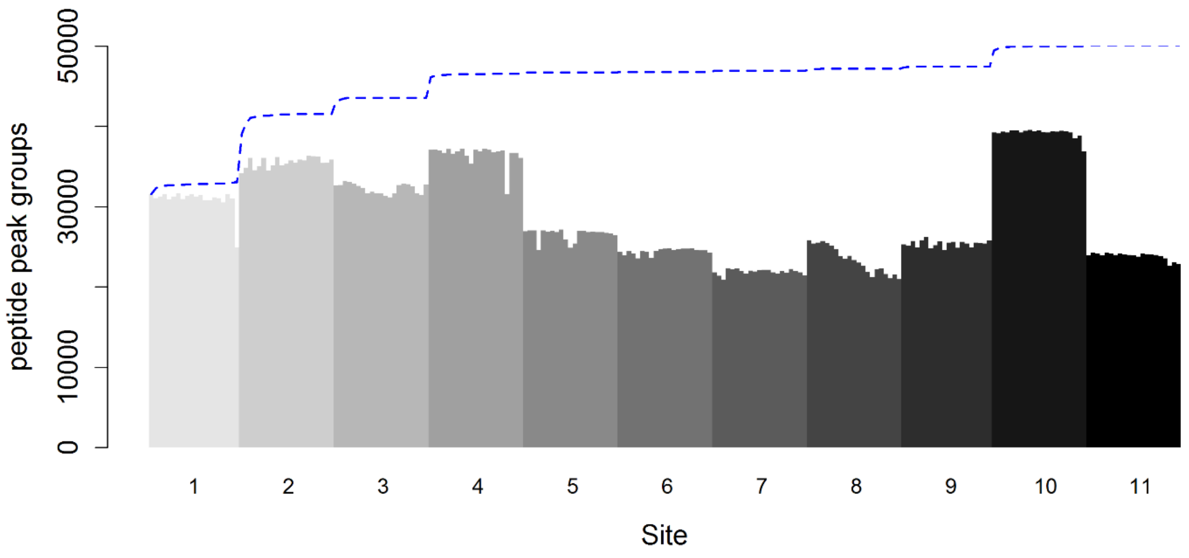

Supplementary Figure 12 – Peptide peak groups detected at 1% FDR – site-by-site analysis

The number of peptide peak groups detected when the data is analysed independently for each site. The data is ordered by site of data collection and then chronologically by time of acquisition. The blue line indicates the cumulate set of proteins detected with each new sample moving from left to right. This is equivalent to Figure 2c, except plotted at peptide peak group level instead of protein level, and also to Supplementary Figure 2 except that the analysis is site-by-site instead of aggregated across sites.

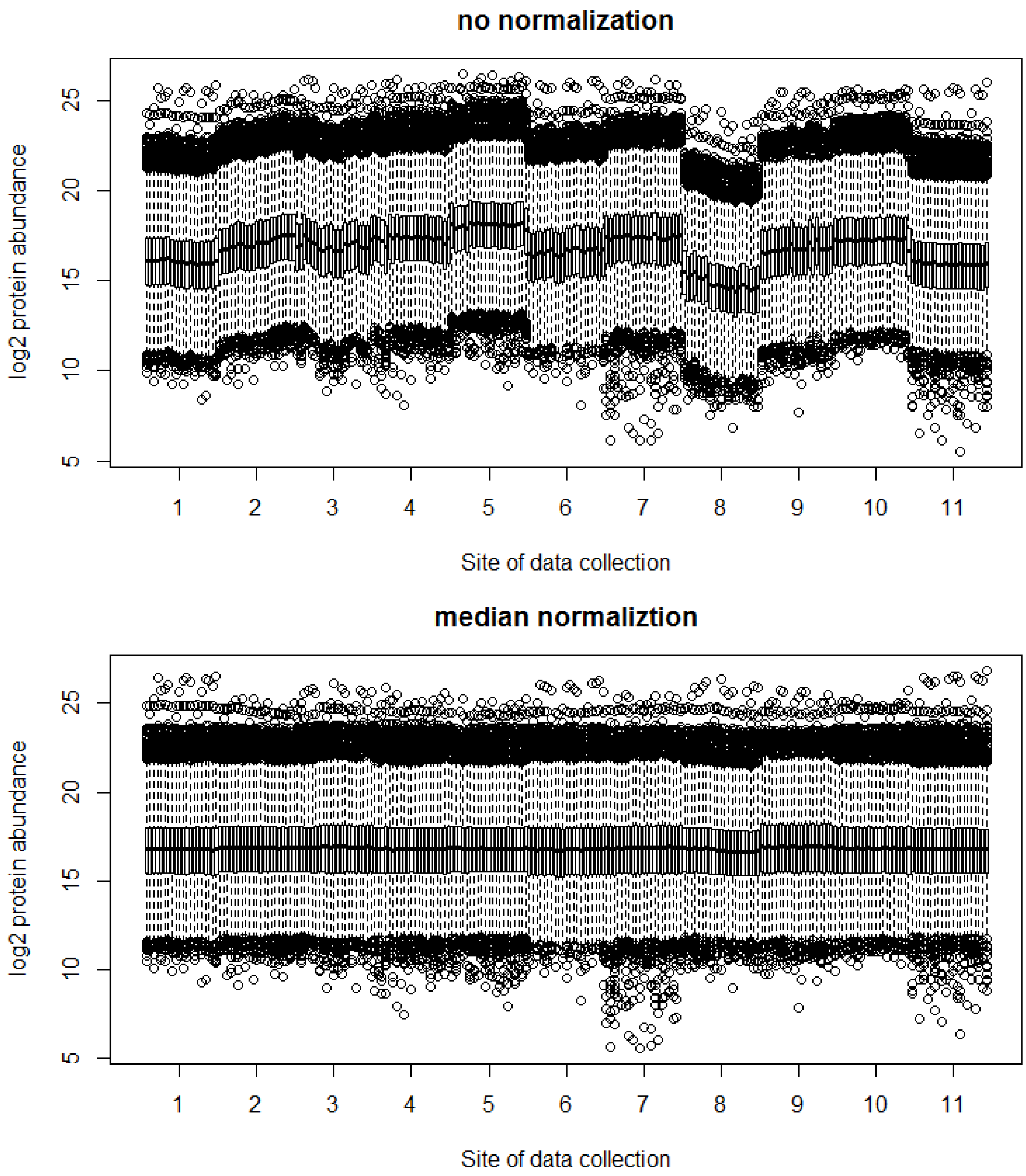

Supplementary Figure 13 – Effect of peptide peak area median normalization on protein abundances

Protein abundances, inferred by summing the top 5 most intense fragment ion peak areas from the top 3 most intense peptide peak groups (or fewer if 3 were not available), are plotted (a) before and (b) after median normalization. The normalization method was to equalize the median peptide peak group area. Boxes indicate the interquartile range with the dividing line indicating the median. The maximum value of the whiskers is 1.5x the interquartile range with values outside the whiskers plotted as circles.

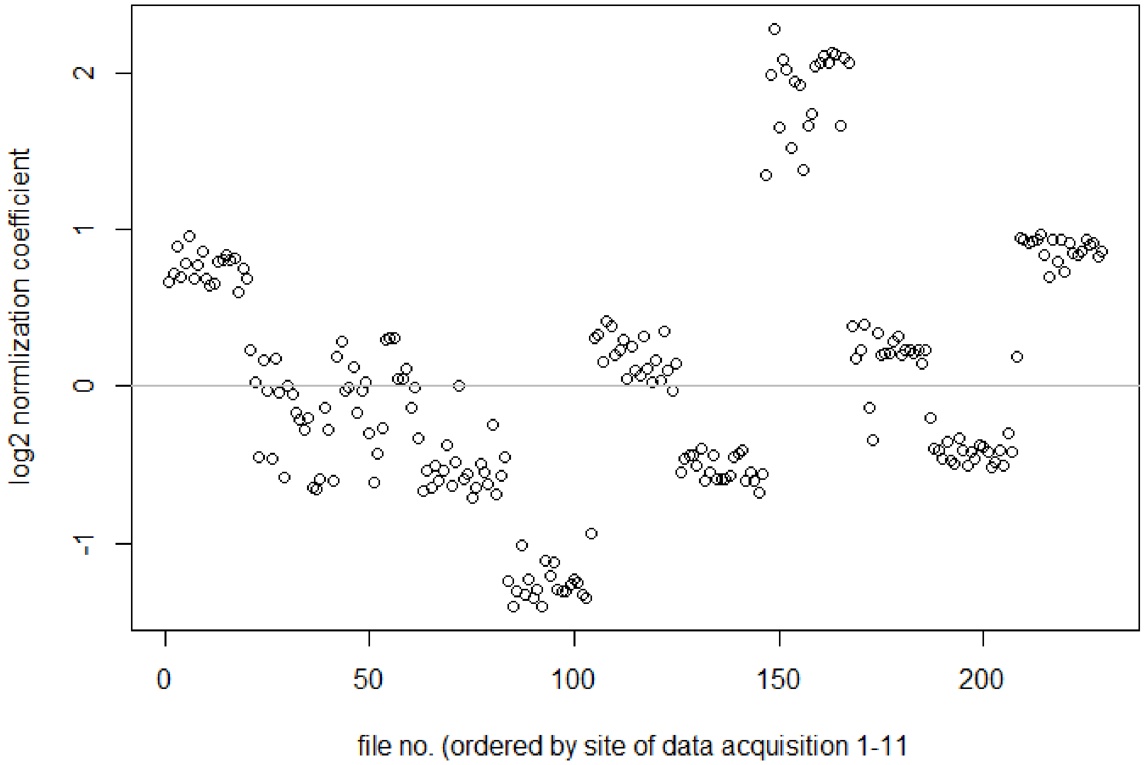

Supplementary Figure 14 – Normalization coefficients used to adjust SIS peptide abundances

Coefficients derived from the normalization procedure for the HEK293 matrix (see Supplementary Figure 13) were also applied to the SIS peptide peak areas. The normalization coefficients are plotted on the log2 scale for easy visualization.

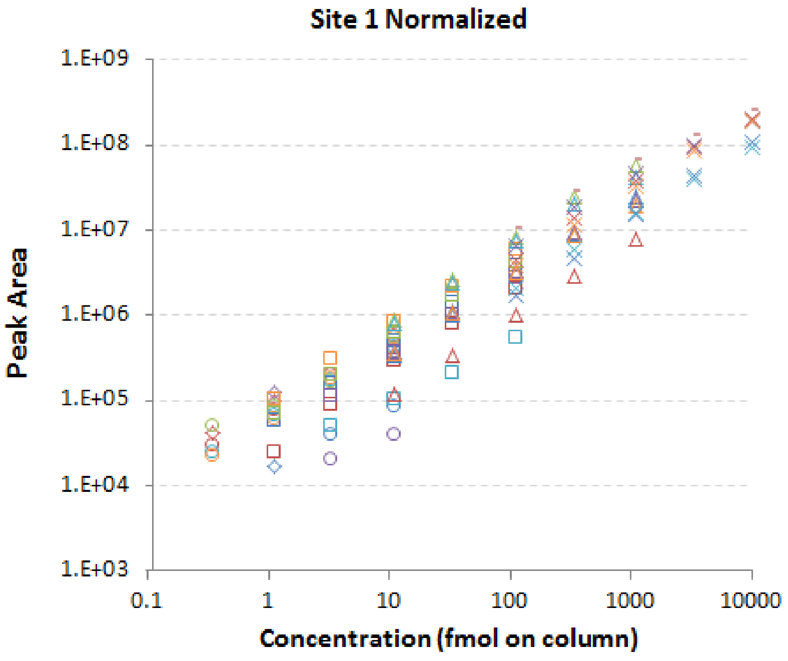

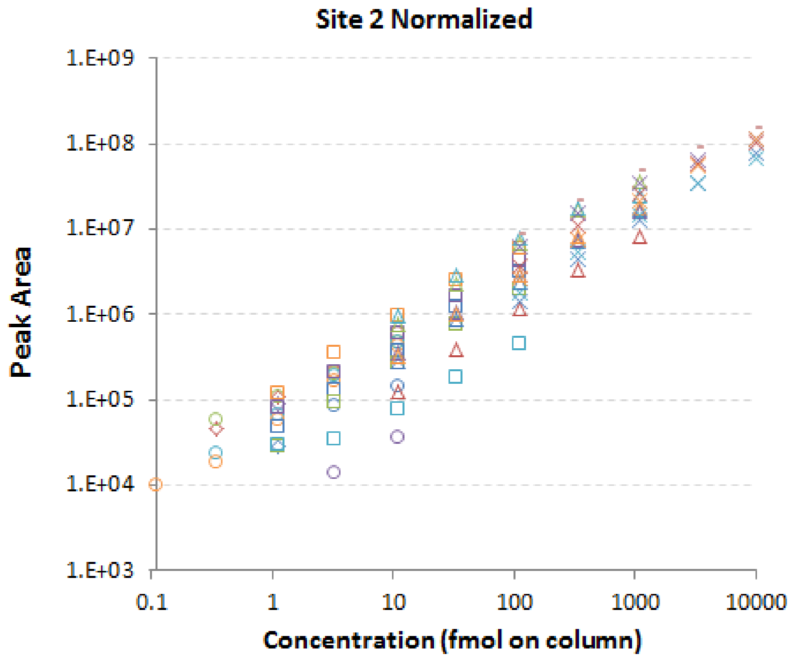

Supplementary Figure 15 – Responses from SIS peptides in dilution series by site

Response curves for summed fragment ion peak areas from SWATH-MS data (as in Fig 4a).

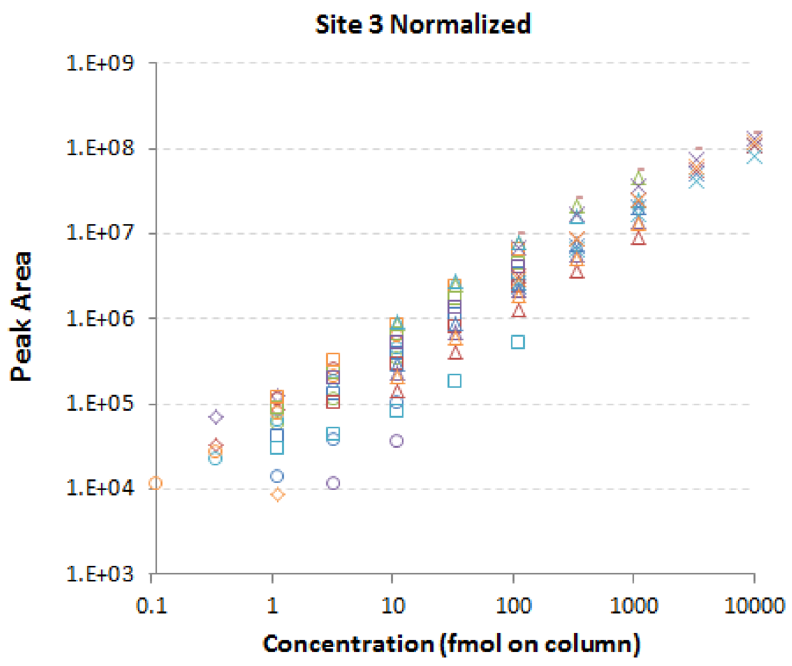

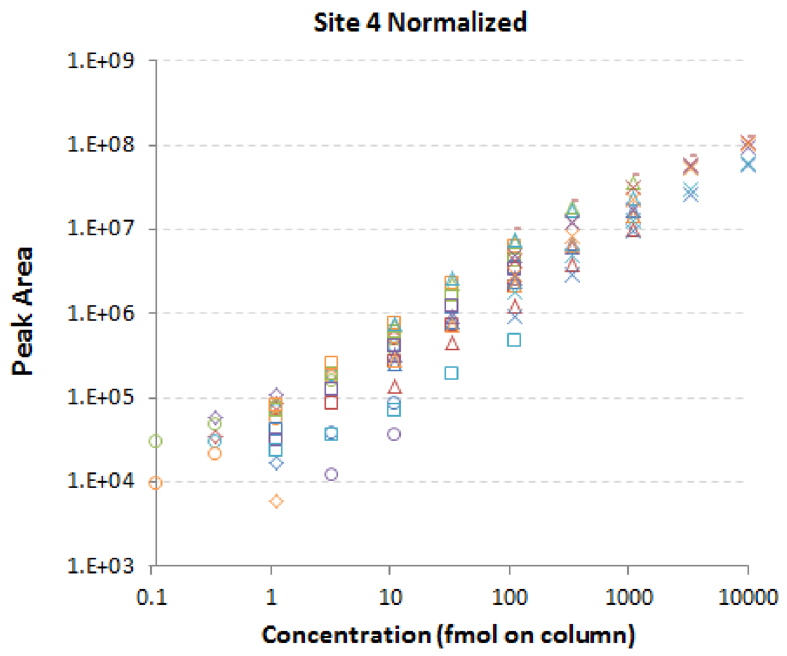

Supplementary Figure 15 (continued) – Responses from SIS peptides in dilution series by site

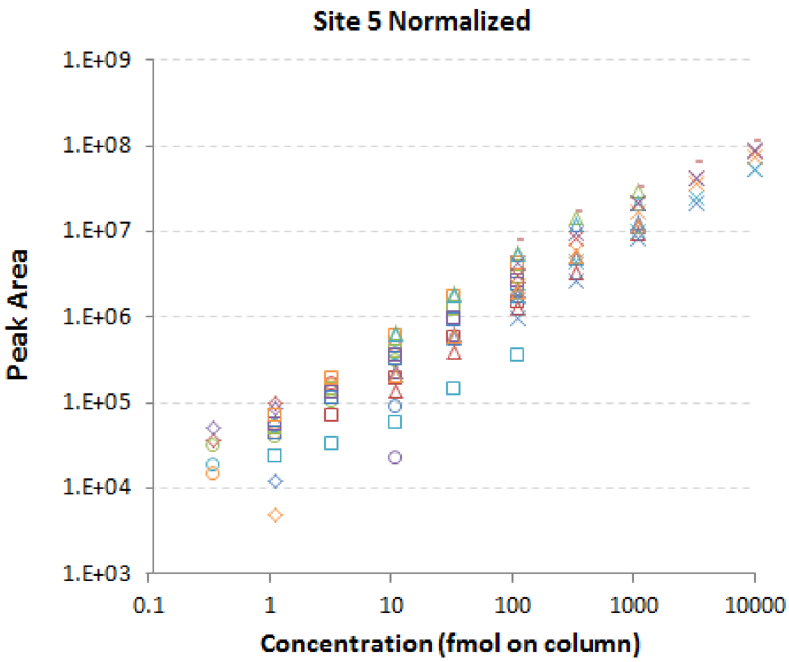

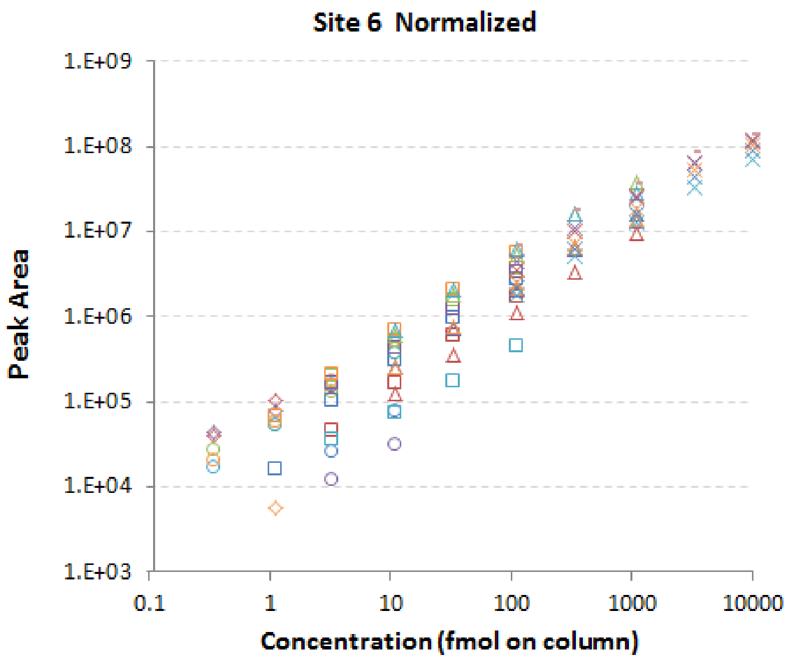

Supplementary Figure 15 (continued) – Responses from SIS peptides in dilution series by site

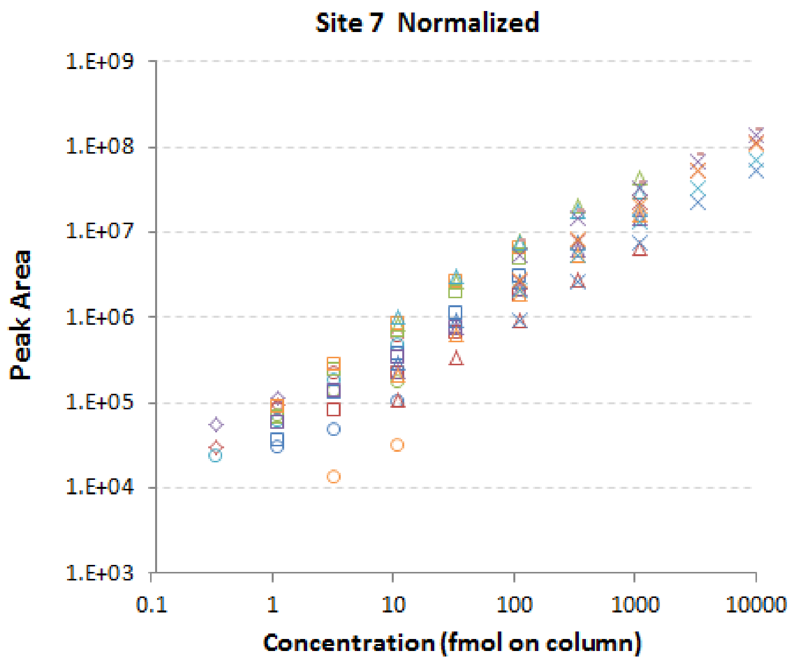

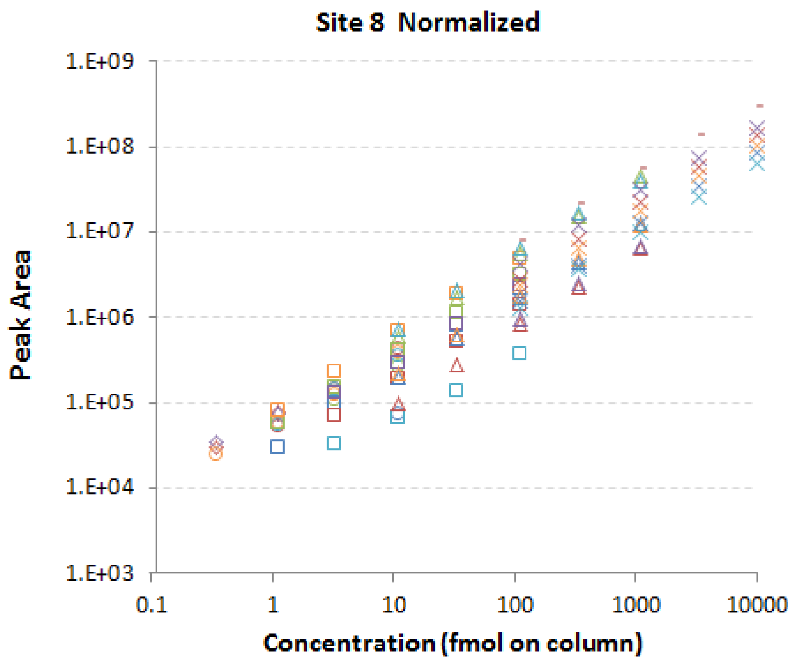

Supplementary Figure 15 (continued) – Responses from SIS peptides in dilution series by site

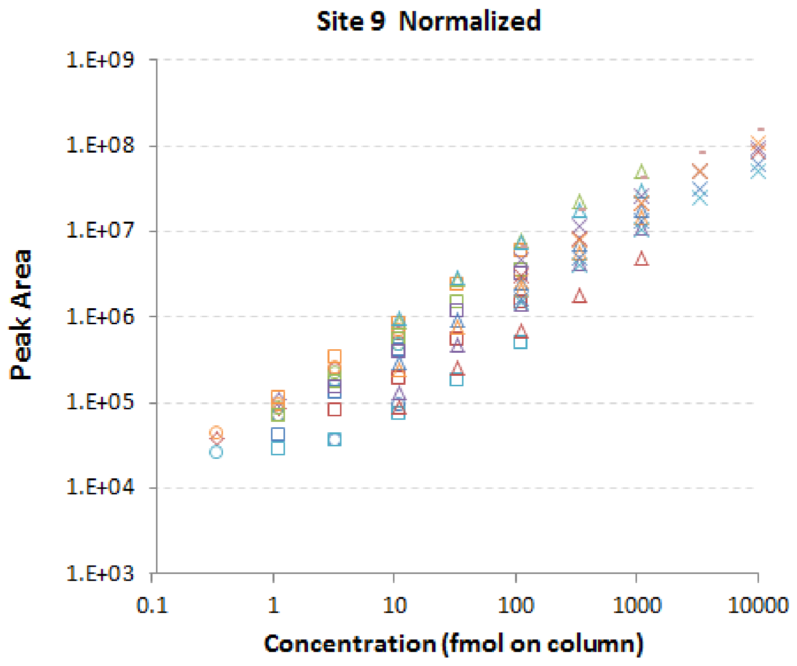

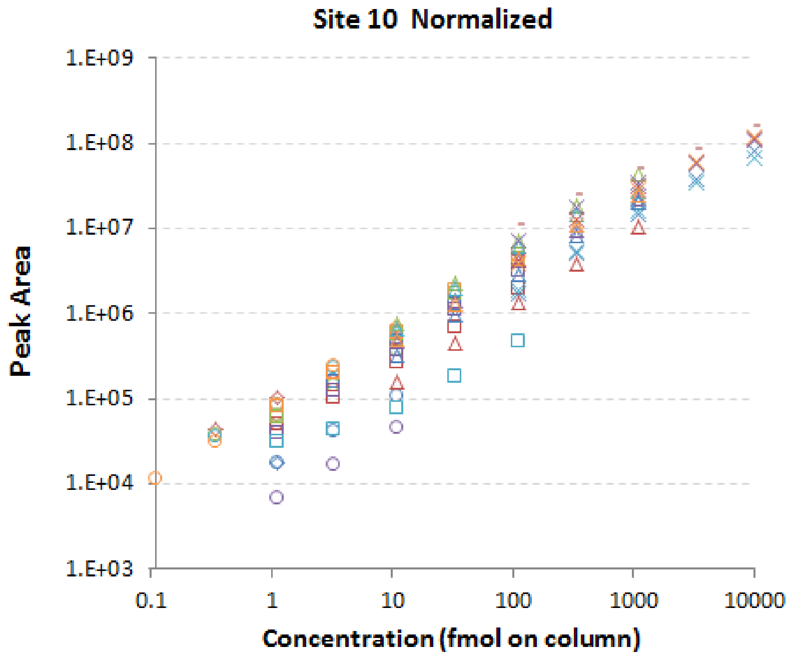

Supplementary Figure 15 (continued) – Responses from SIS peptides in dilution series by site

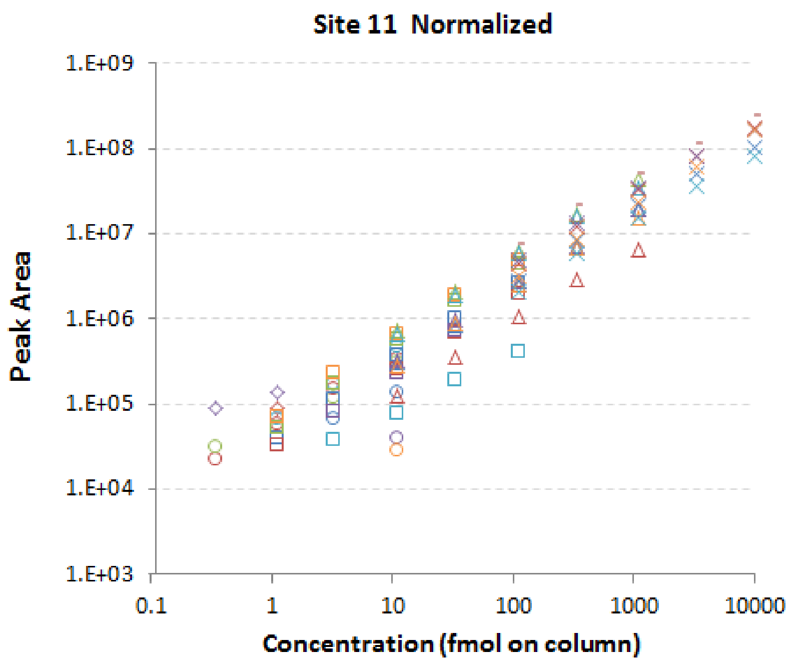

Supplementary Figure 15 (continued) – Responses from SIS peptides in dilution series by site

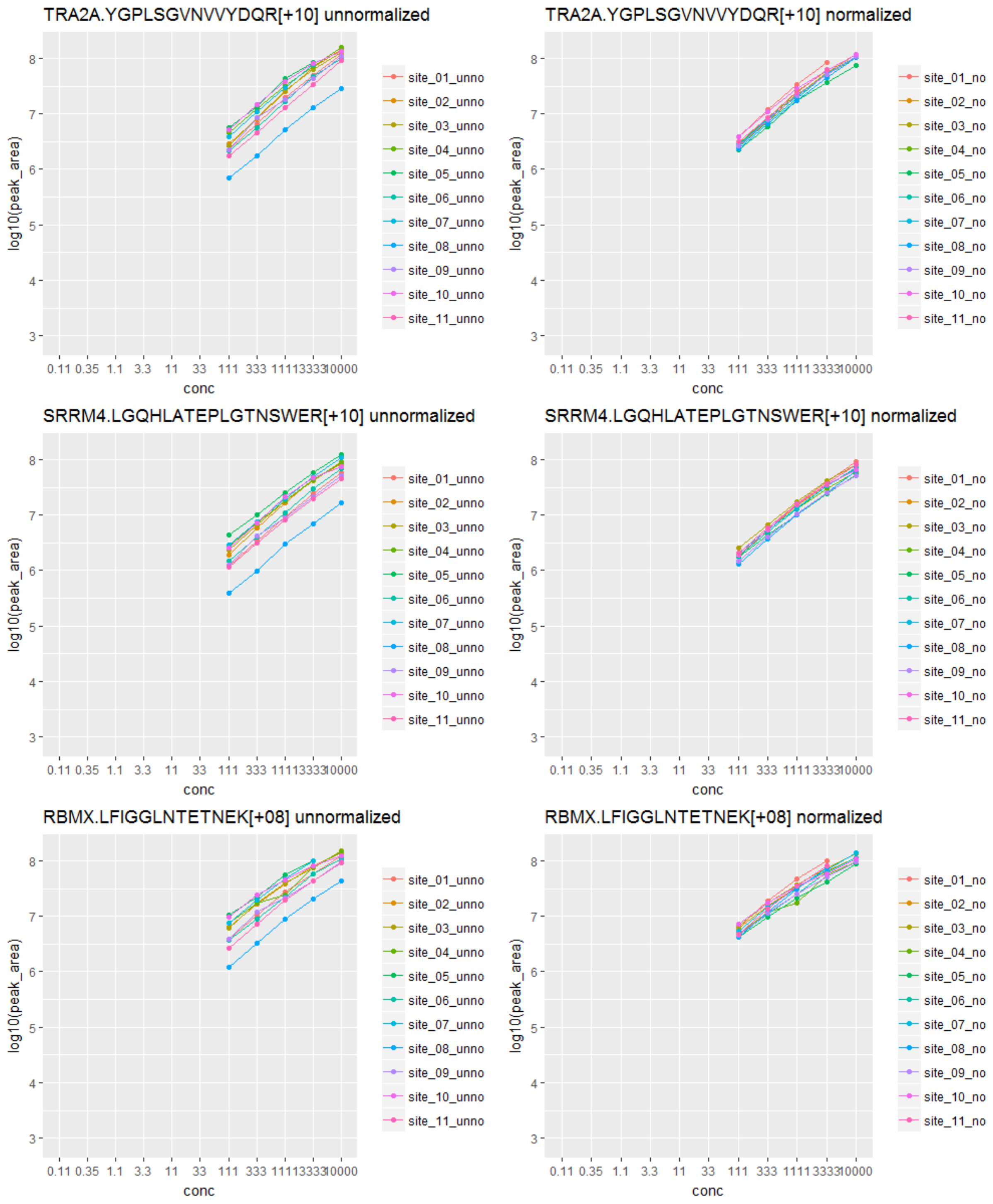

Supplementary Figure 16 – Responses from SIS peptides in dilution series by peptide

As in Supplmentary Figure 15 except separated by peptide instead of site.

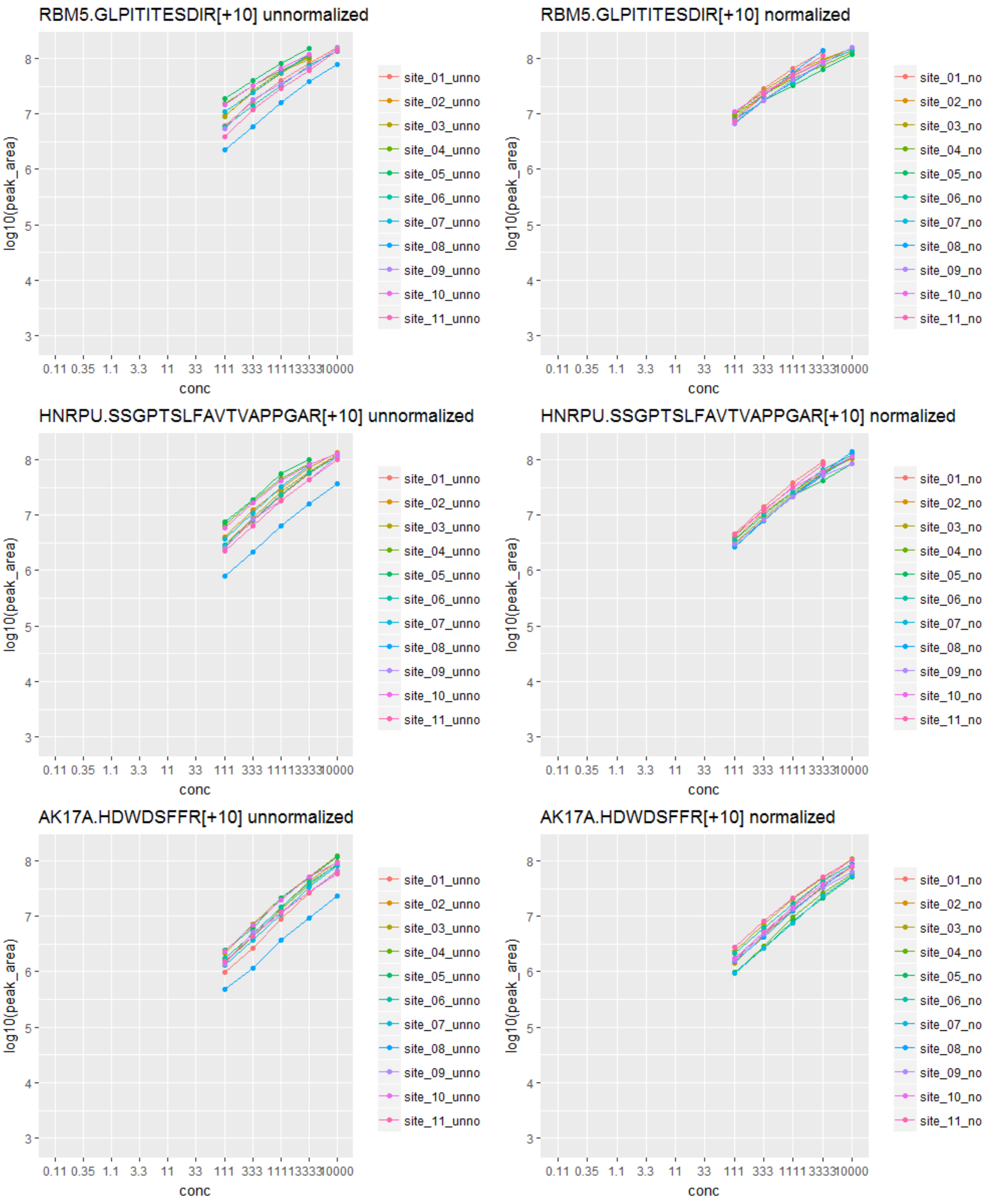

Supplementary Figure 16 (continued) – Responses from SIS peptides in dilution series by peptide

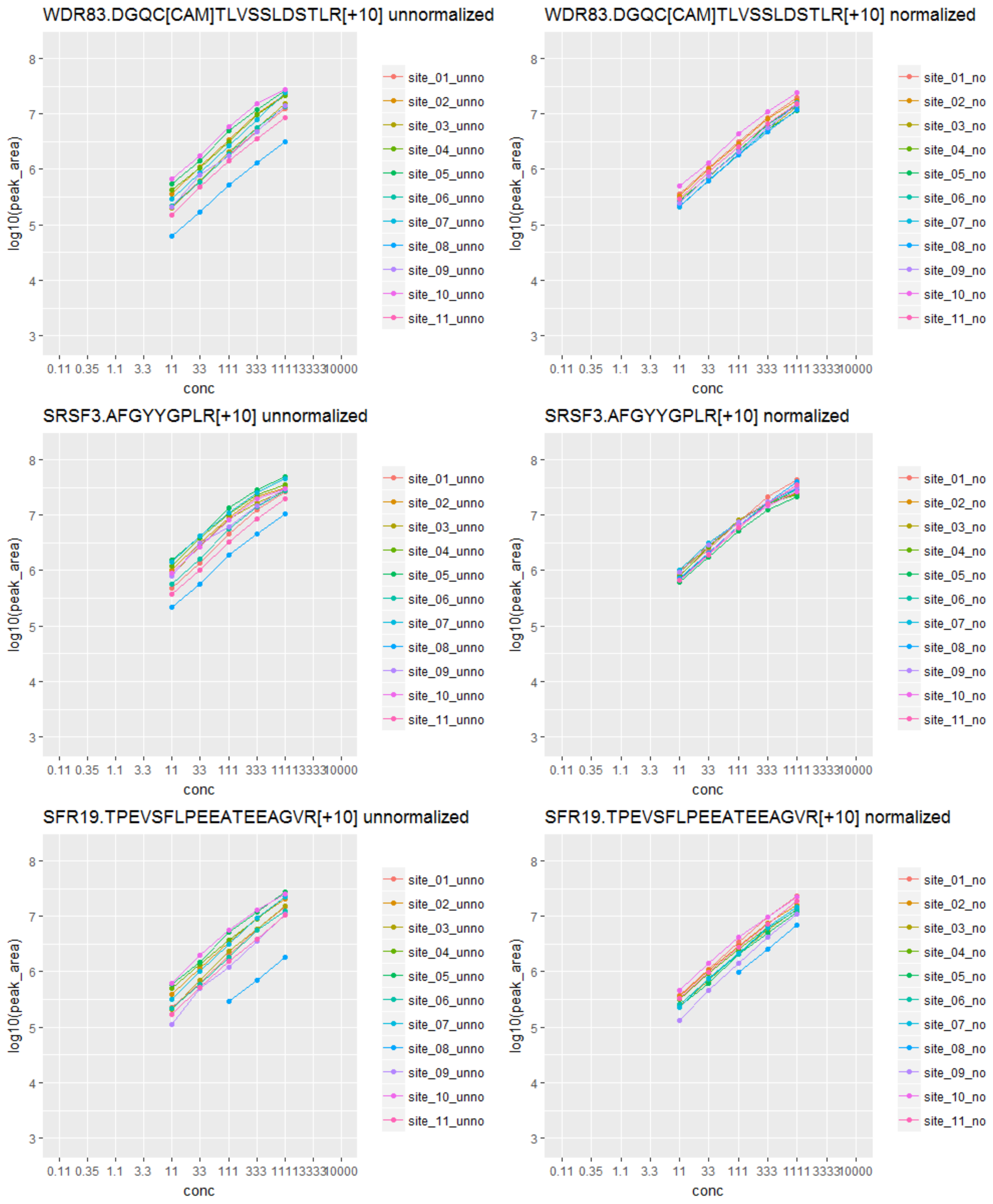

Supplementary Figure 16 (continued) – Responses from SIS peptides in dilution series by peptide

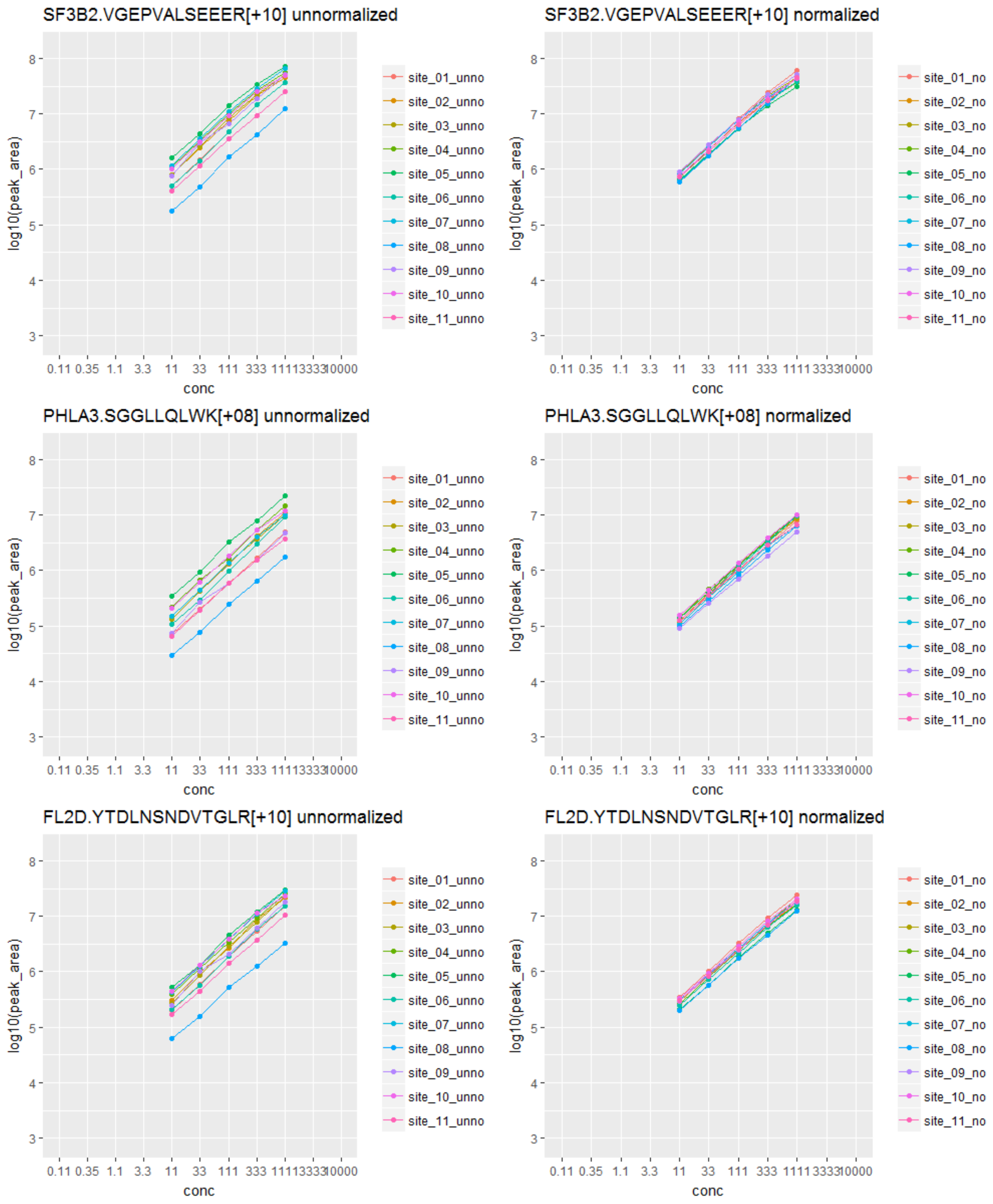

Supplementary Figure 16 (continued) – Responses from SIS peptides in dilution series by peptide

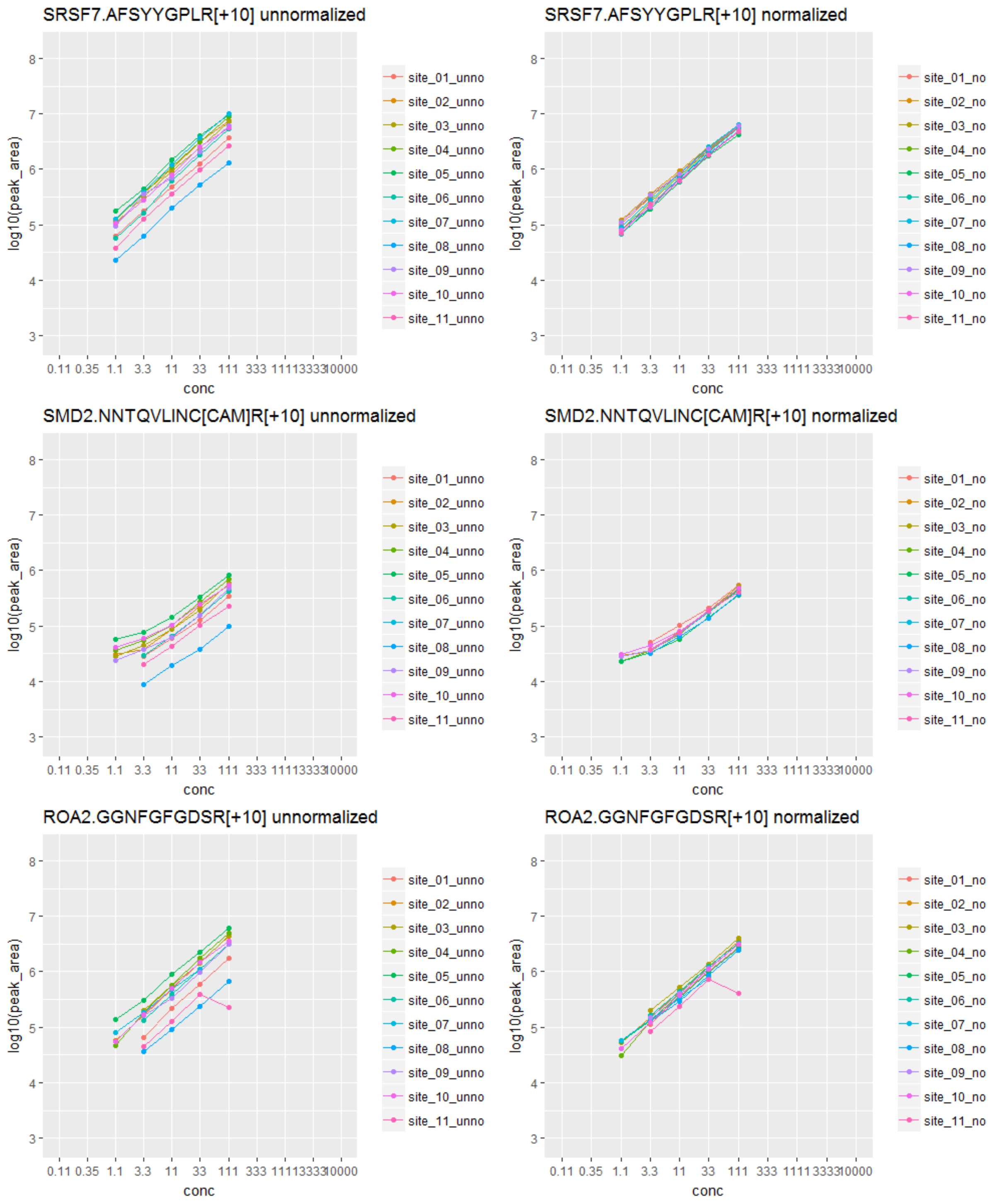

Supplementary Figure 16 (continued) – Responses from SIS peptides in dilution series by peptide

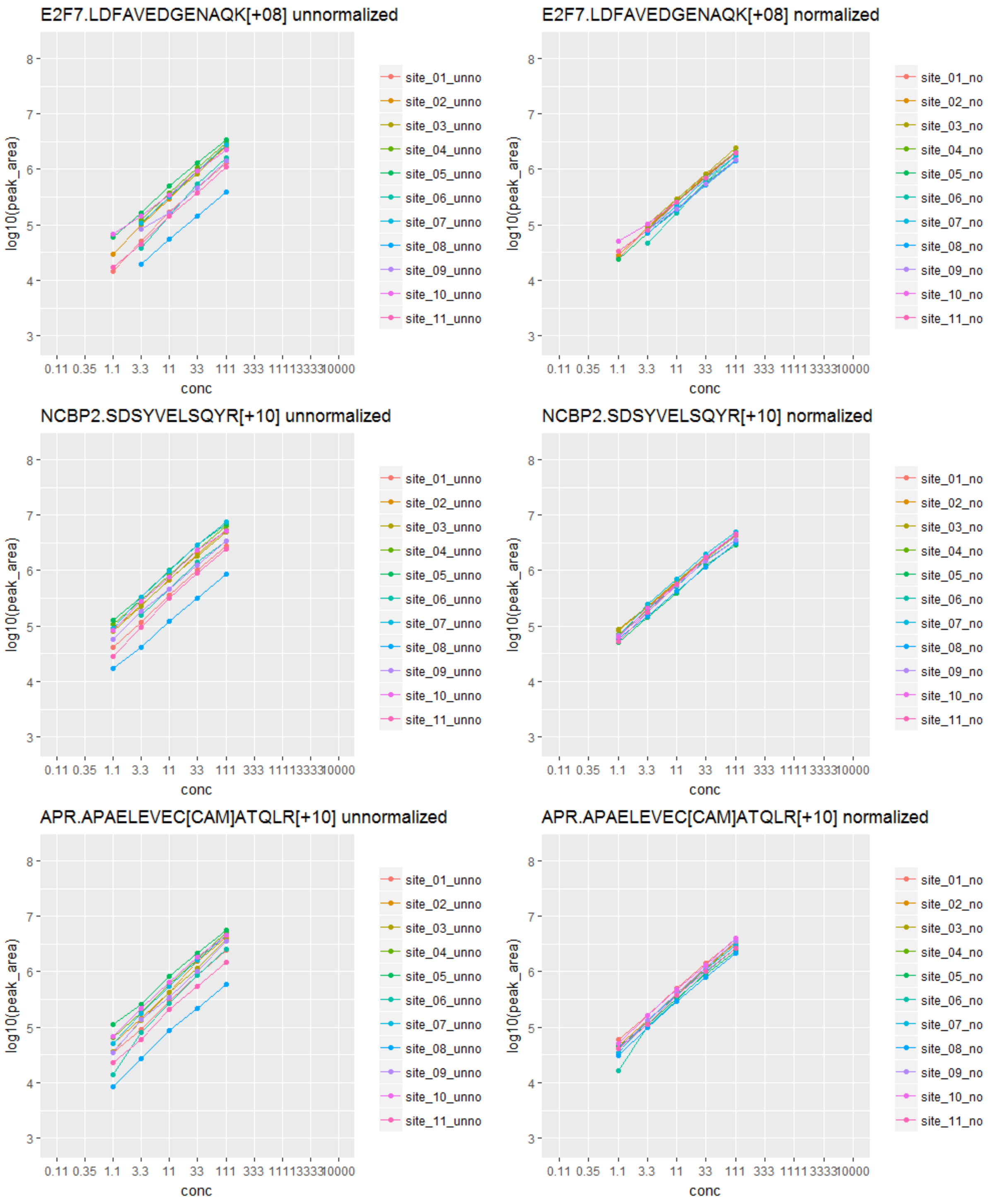

Supplementary Figure 16 (continued) – Responses from SIS peptides in dilution series by peptide

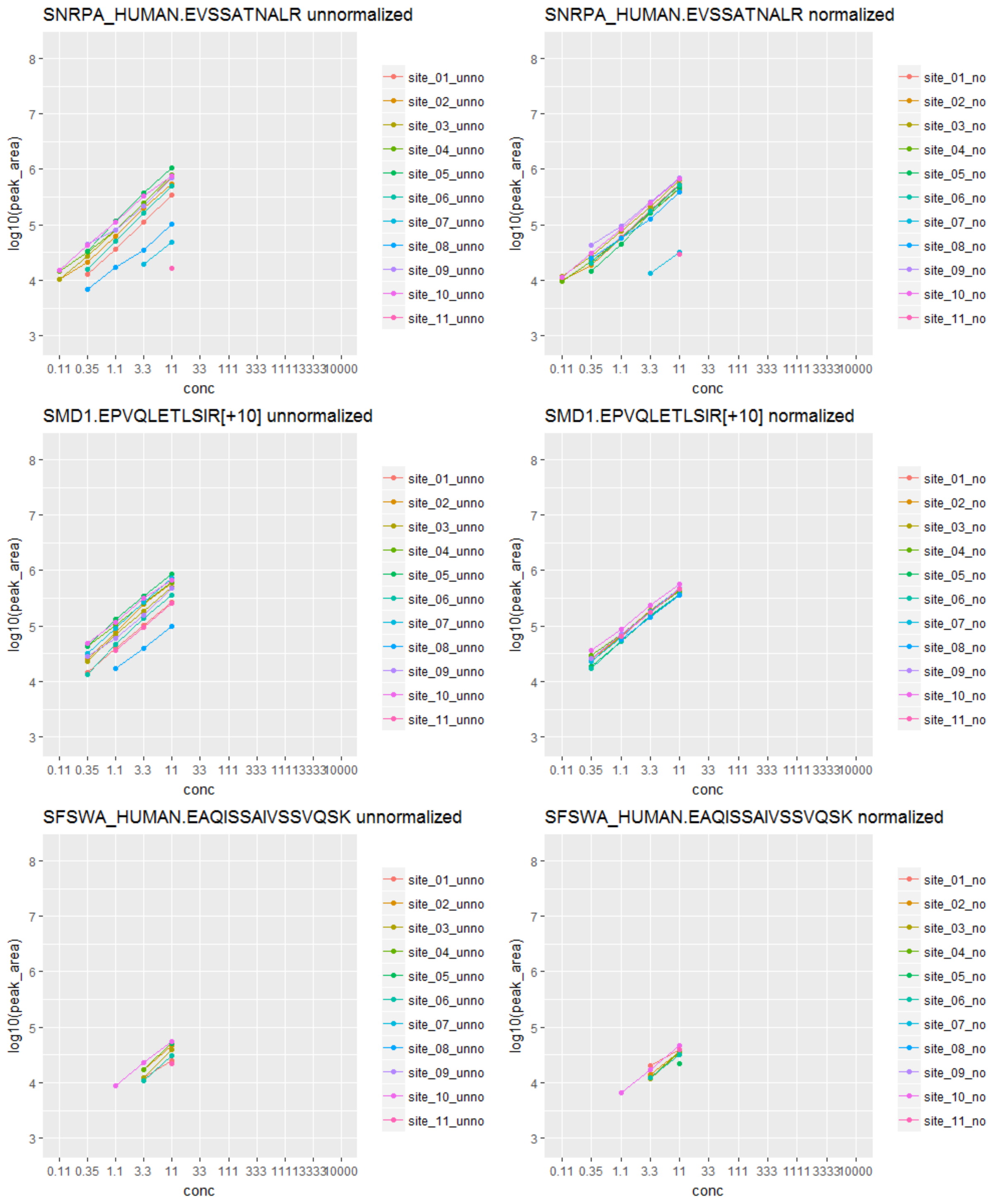

Supplementary Figure 16 (continued) – Responses from SIS peptides in dilution series by peptide

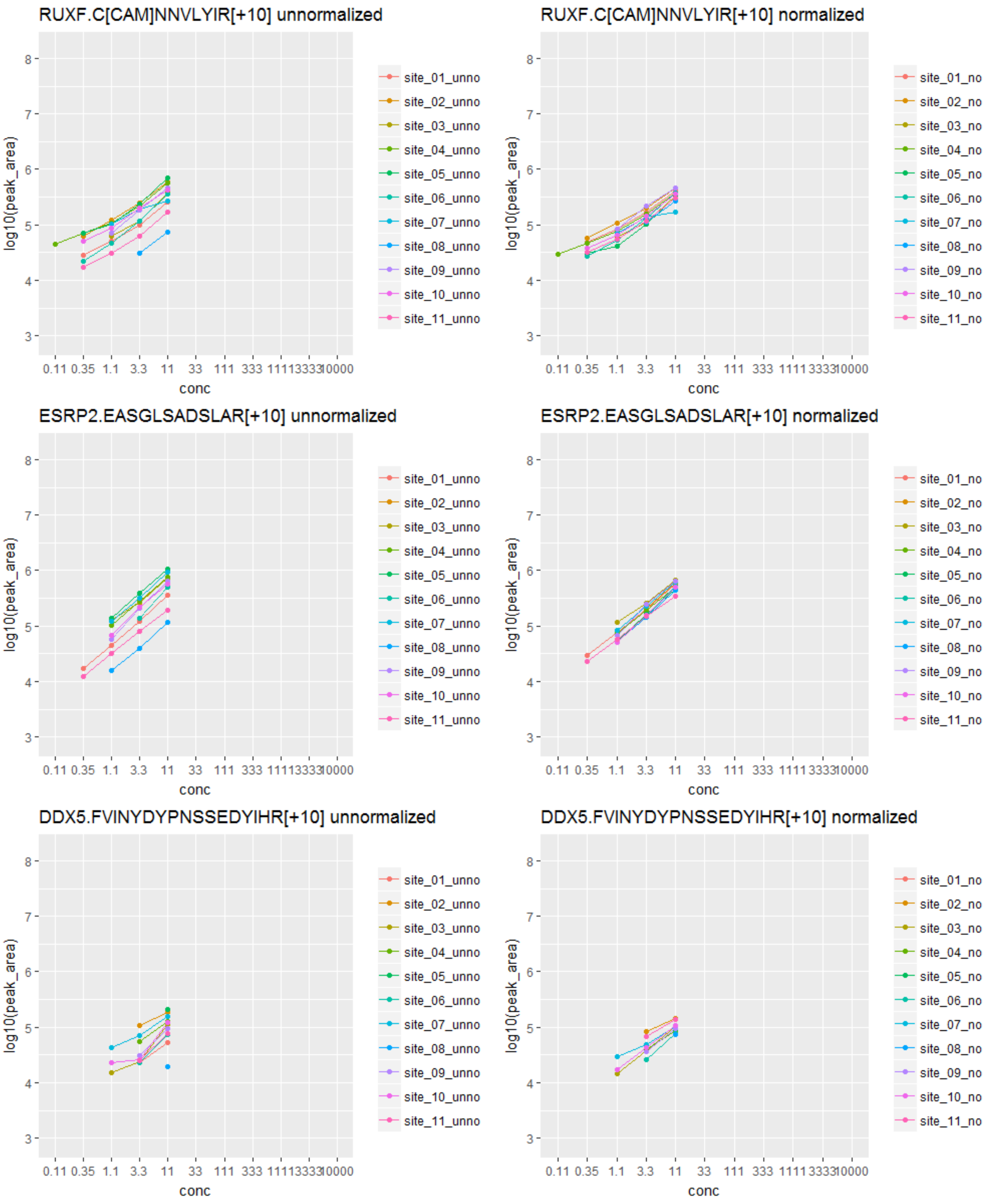

Supplementary Figure 16 (continued) – Responses from SIS peptides in dilution series by peptide

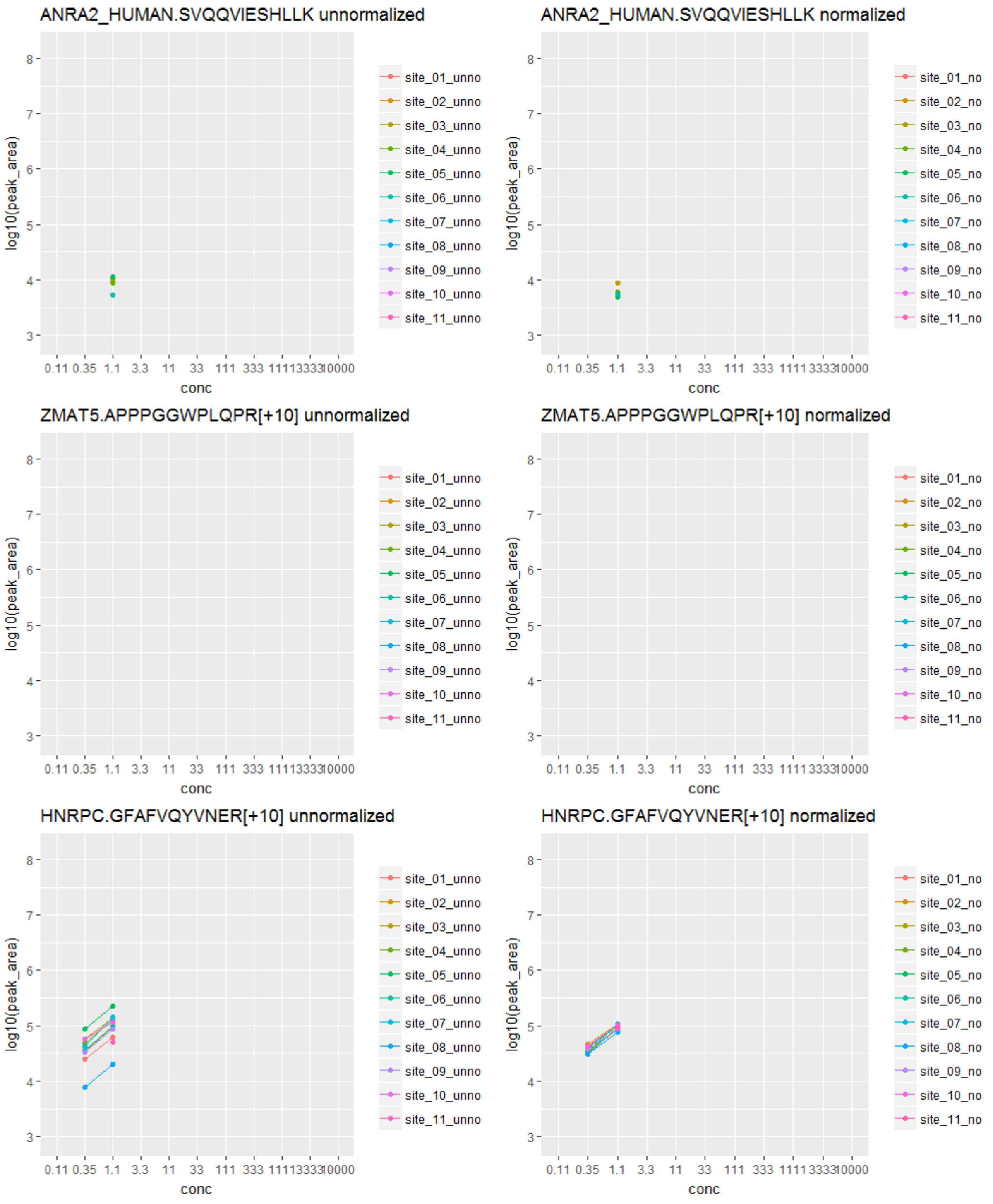

Supplementary Figure 16 (continued) – Responses from SIS peptides in dilution series by peptide

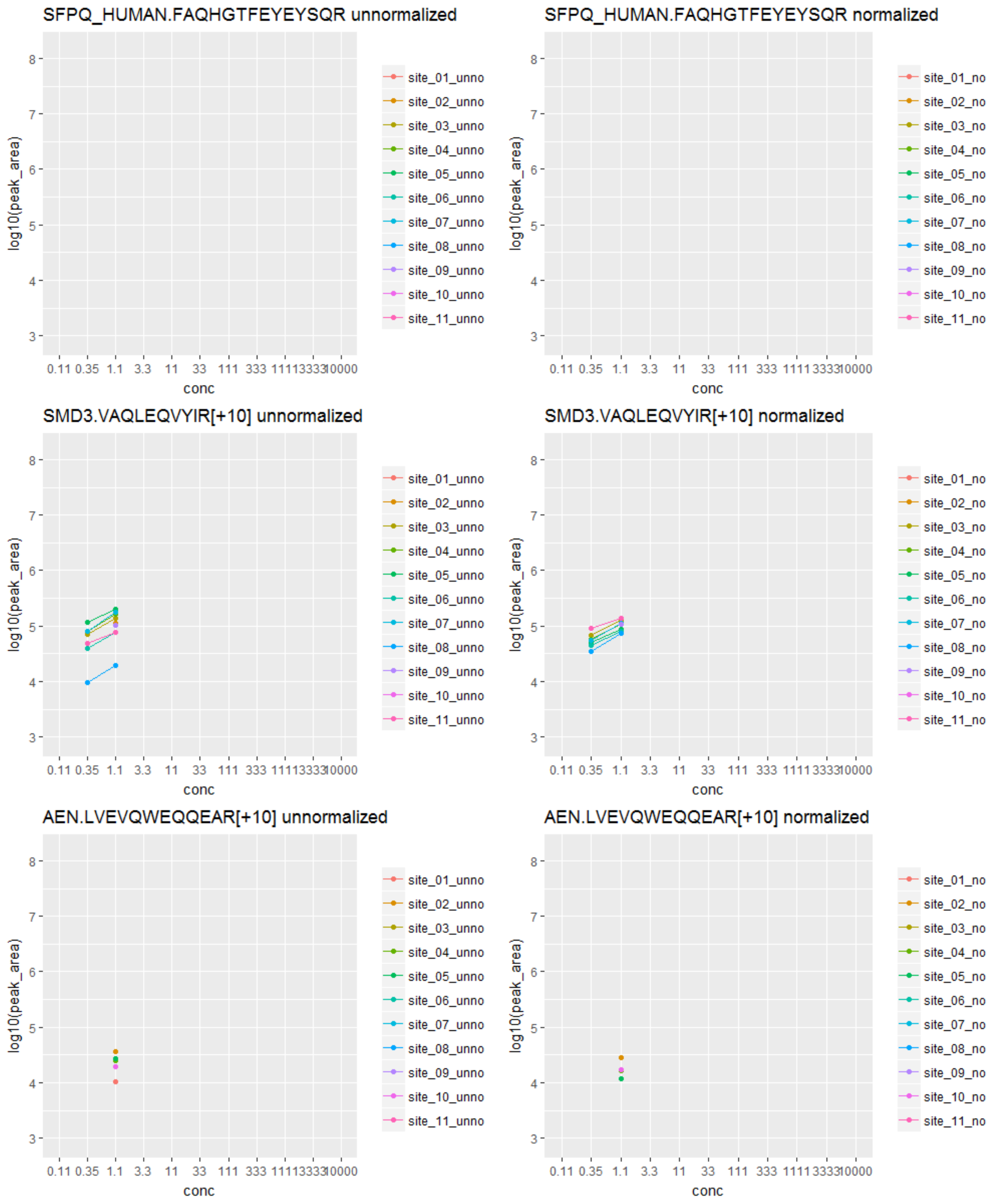

Supplementary Figure 16 (continued) – Responses from SIS peptides in dilution series by peptide

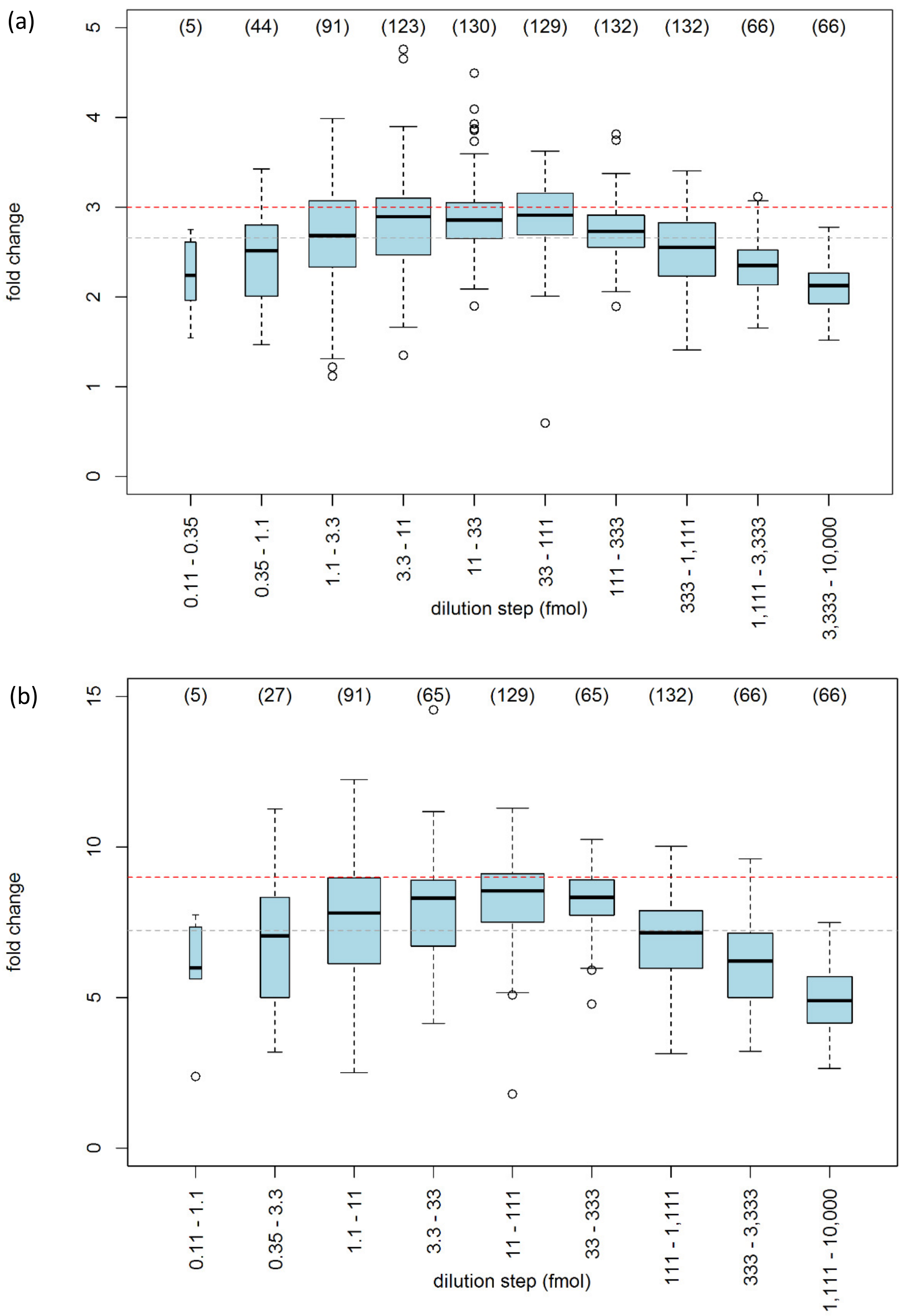

Supplementary Figure 17 – Measured versus expected fold change of SIS peptides

The distribution of fold changes of SIS peptides is shown. The expected fold change (3-, 9-, 27-, and 81-fold in panels a, b, c, and d respectively as computed by comparing different points across the SIS peptide concentrations) is indicated by the dashed red line and the average measured fold change (2.66, 7.49, 19.6, 52.4 respectively) is indicated by the dashed grey line. Replicate measurements with one site at a given concentration point are averaged such that the number of data points per concentration (indicated in brackets) includes the various SIS peptides measured at various sites. As with the dynamic range curves we include data for LLOQ and above. Boxes indicate the interquartile range with the dividing line indicating the median.

The maximum value of the whiskers is 1.5x the interquartile range with values outside the whiskers plotted as circles. The dilution steps are nominal values with the exact values given in Supplementary Data 1.

(c)

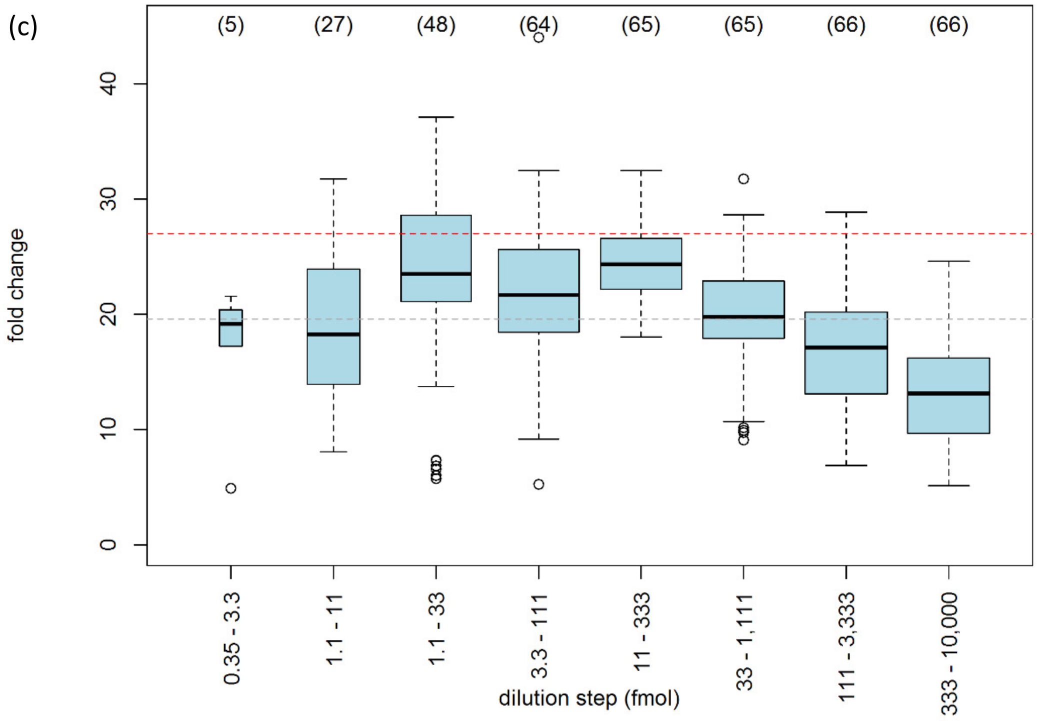

(d)

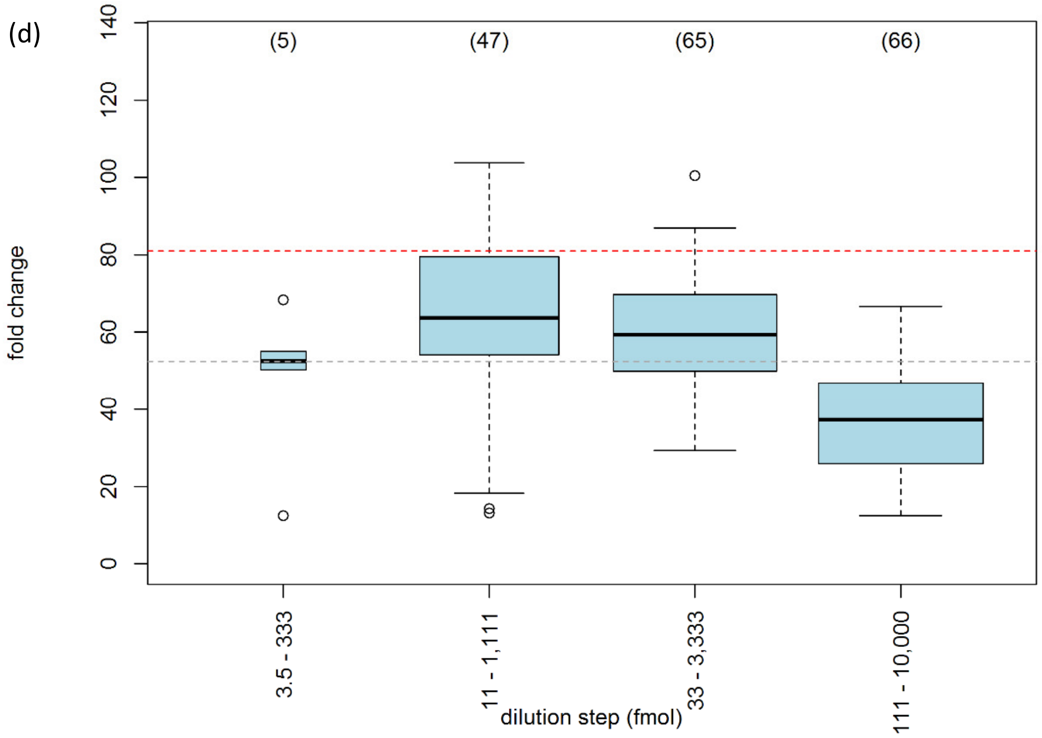

Supplementary Figure 17 (cont'd) – Measured versus expected fold change of SIS peptides

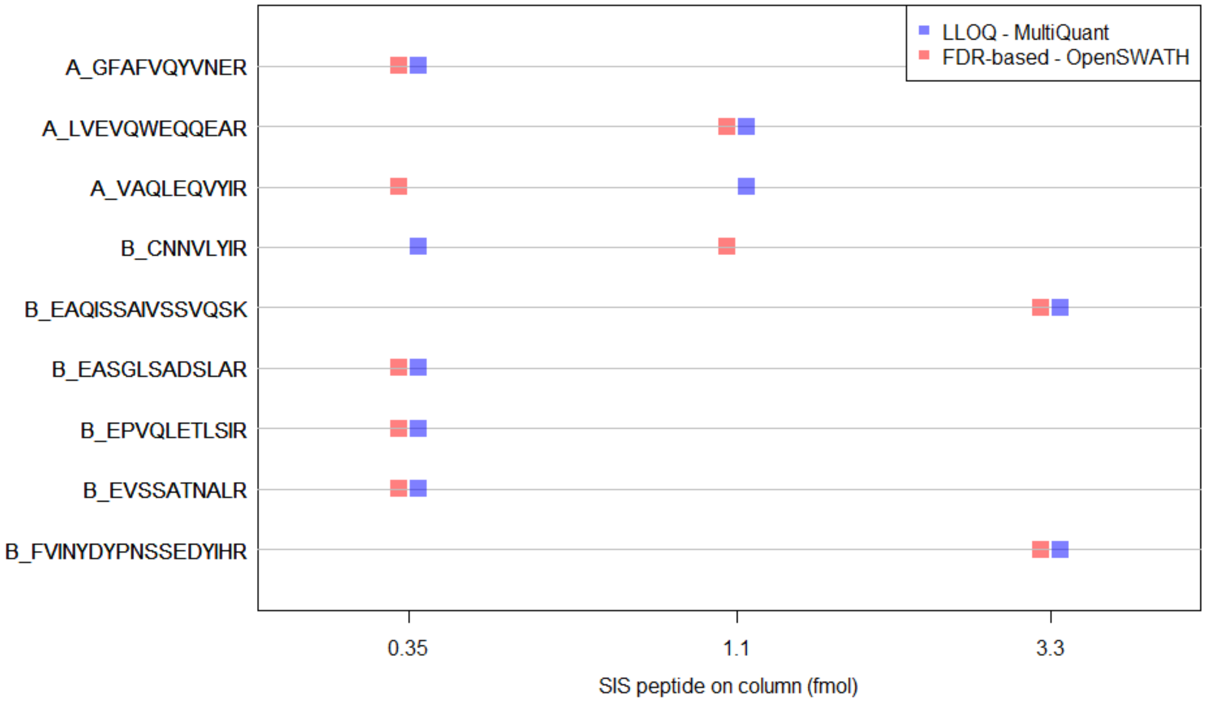

Supplementary Figure 18 – Comparison of MultiQuant vs. OpenSWATH assessment of SIS peptides – LLOQ vs FDR based filtering

The LLOQ (lower limit of quantification) as determined by MultiQuant analysis (<20% CV, 80-120% accuracy, and S/N>20 and manual inspection) is compared with the lowest concentration at which the peptide could be detected using the FDR based and automated OpenSWATH analysis for peptides from groups A and B which span the lower concentration ranges from low attomole to low femtomole. Representative data from site 1 is shown.

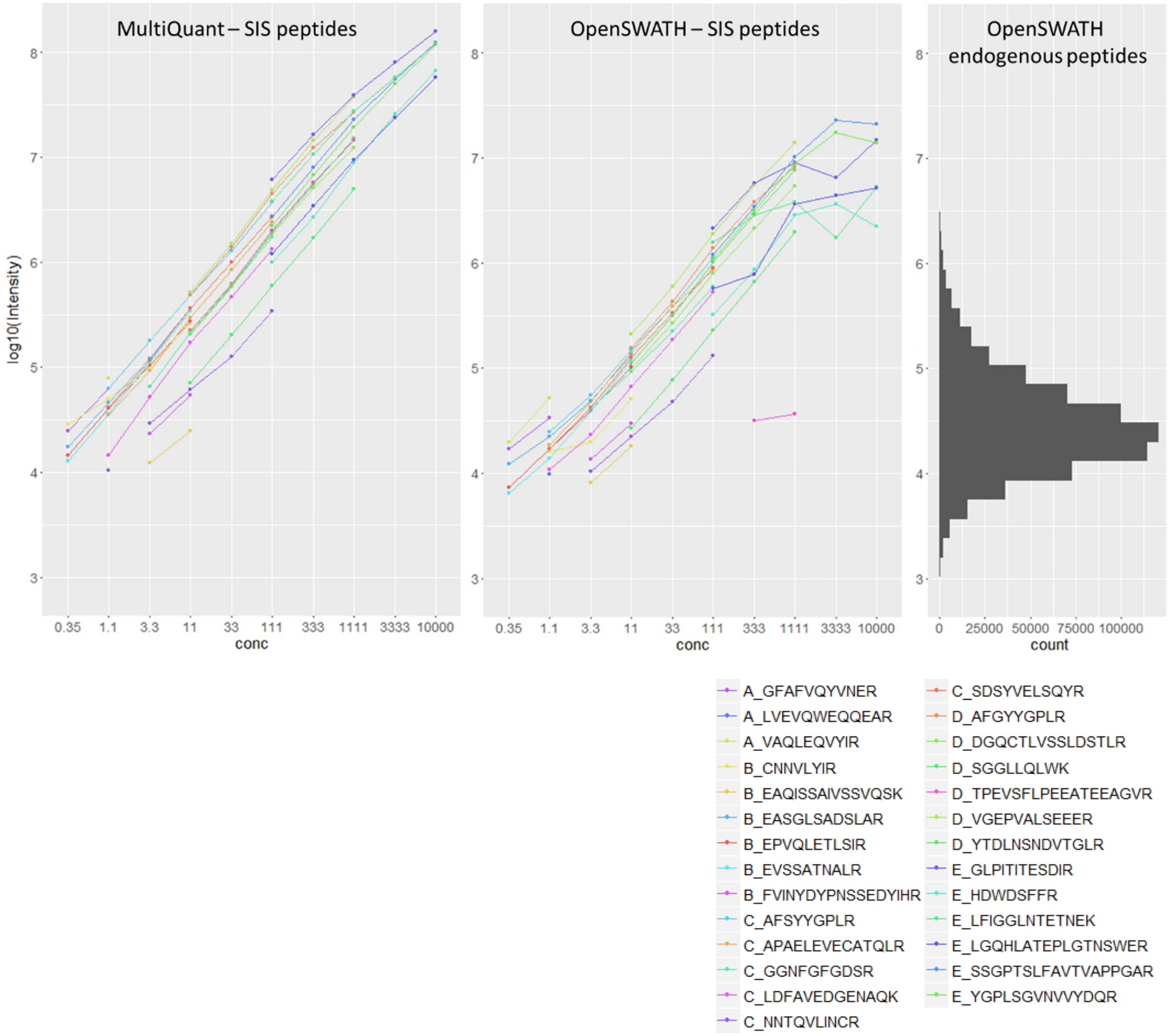

Supplementary Figure 19 – Comparison of MultiQuant vs. OpenSWATH assessment of SIS peptides – Linear dynamic range

The dilution curves displaying linearity and dynamic range for SIS peptides as determined by MultiQuant analysis (left panel) is compared with that achieved by the automated OpenSWATH analysis (middle panel). The distribution of endogenous peptide intensities from the HEK293 proteome is shown for reference on the same scale (right panel). Representative data from site 1 is shown.

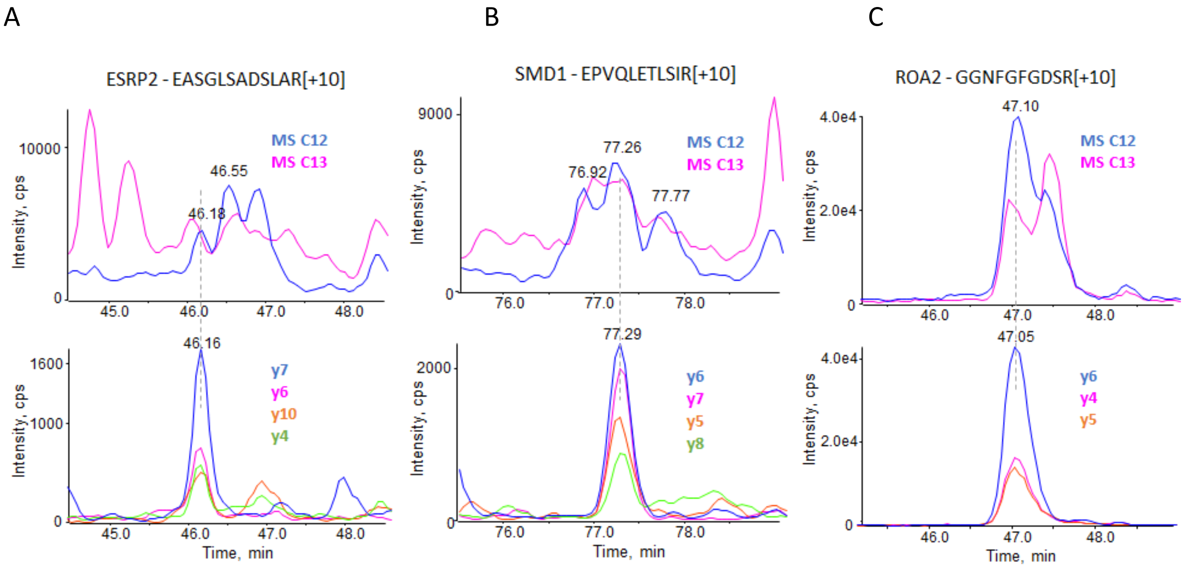

Supplementary Figure 20 – Examples of typical S/N differences between MS1 and SWATH-MS data

(A) The data from an S3 sample acquisition at Site 1 for the heavy isotope labelled peptide EASGLSADSLA**R[+10]** peptide (ESRP2 protein) at 1.11 fmol on column in HEK matrix is shown, for both the MS and SWATH-MS data. This concentration is the LLOQ for MS1 of 1.11 fmol. The corresponding SWATH-MS data was much cleaner and therefore could be confidently quantified at a lower concentration, with an LLOQ of 0.37 fmol on column. (B) Example from the S3 sample acquisition at Site 2, the heavy isotope labelled peptide EPVQLETLSI**R[+10]** peptide (SMD1 protein) for the 1.11 fmol on column point, where interferences are seen in the MS1 data. For this peptide at this site, the LLOQ for the MS1 data was 3.3 fmol on column whereas the SWATH-MS data LLOQ was 0.37 fmol. (C) Interferences are possible in the SWATH-MS based data, however with multiple fragments per peptide to choose, it is possible to find a set of fragments that provide clean quantification with a good LLOQ. For Site 3, the heavy isotope labelled peptide GGNFGFGDS**R[+10]** (ROA2 protein) had a small amount of interference in the 4th highest fragment ion, so the top 3 were used providing clean signal and an LLOQ of 3.7 fmol on column. The MS1 data had a significant interference in both isotopes, as shown here for the injection of 33.3 fmol on column, hence the MS1 LLOQ was 100 fmol.

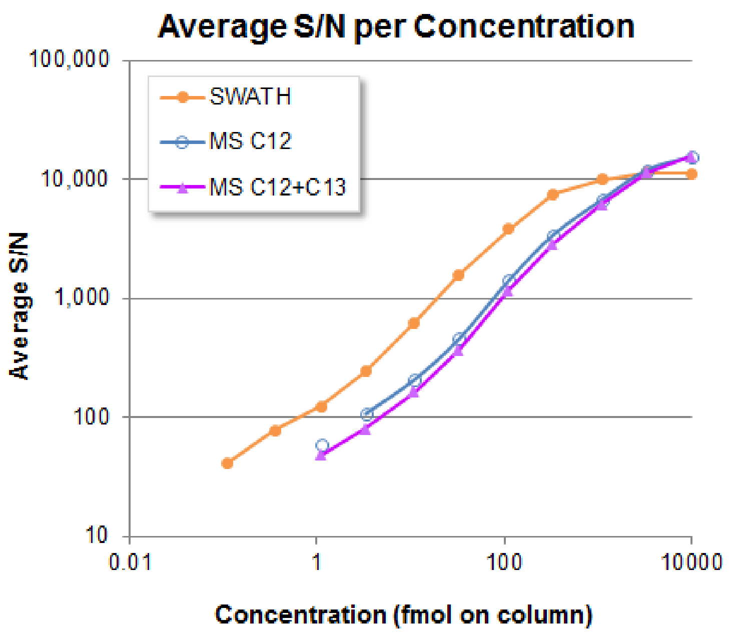

Supplementary Figure 21 – Average S/N observed across the concentration curve

The S/N for each XIC peak at each concentration was determined and therefore could be averaged across peptides for a particular concentration. This was done on the summed SWATH-MS data as well as the MS1 data, for the C12 data and the C12+ C13 summed data. The S/N observed (orange) for the SWATH-MS data was found to be significantly higher than the S/N for the MS level data for each peptide at most peptide concentrations (C12 data in blue). A further small decrease in average S/N was observed when the second isotope (C12 + C13 data in purple) was summed into the MS1 data.

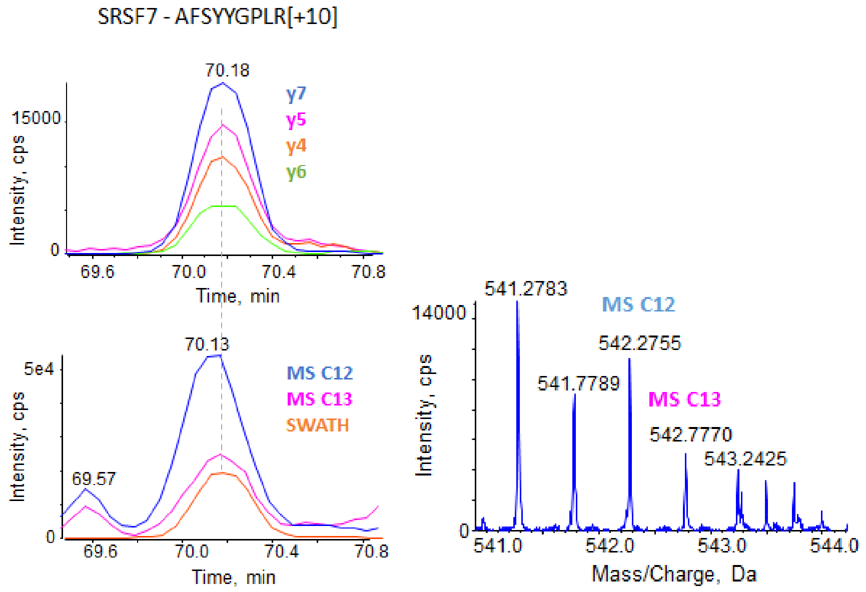

Supplementary Figure 22 – Assessing interferences in C12 vs C13 isotopes of MS1 data

Interferences and their consequences exemplified on the heavy isotope labelled peptide AFSYYGPL**R[+10]** from the Site 2 data set. The data indicate that the C12 peak had a low level interference that became visible at lower concentrations. Shown here is the SWATH-MS data (top) and the MS1 data (bottom) for this peptide in Sample 3, 11 fmol of peptide on column. The summed SWATH-MS trace (orange) is included on the MS1 peak to help with the visualization of peak shape. Notice that the C12 trace (blue) is shifted to the left relative to the C13 (pink) and SWATH-MS. Viewing the underlying full scan MS1 spectra at the peak apex shows that there is a nearly co-eluting doubly charged peptide that is only 2 m/z different in mass. The second C13 isotope from this peptide contributes to the C12 signal of the target peptide at the lower concentrations of the curve, impacting quantification. As the sequence of this co-eluting peptide is different, the resulting fragment ions are different and therefore the SWATH data remains interference-free.

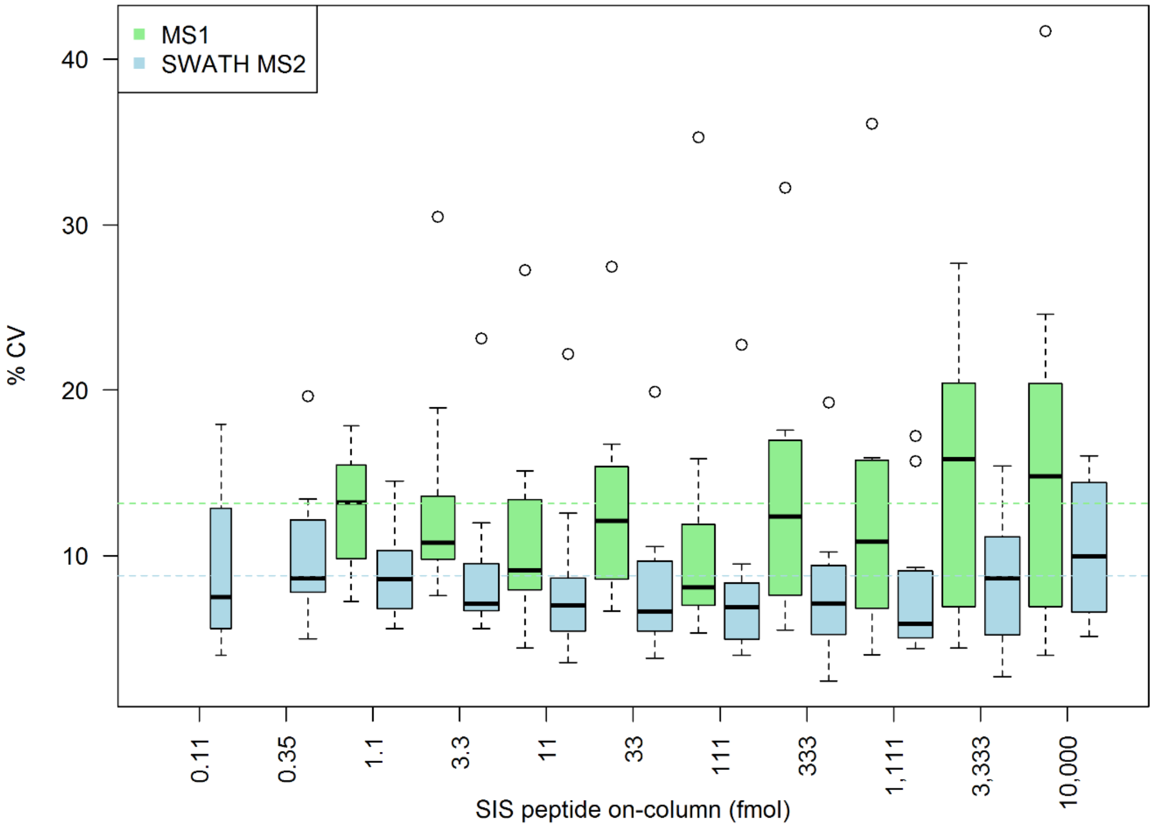

Supplementary Figure 23 – Reproducibility of SWATH-MS versus MS1 quantification

The intra-lab CVs, binned by concentration level, are compared for MS1 (green) and SWATH-MS (blue) quantification. Dashed lines indicate the overall medians for MS1 and SWATH-MS, with the intra-lab reproducibility (%CV) for SWATH-MS and MS1 of 8.8% and 13.2%, respectively.

Supplementary Method 1 - Standard Operating Procedure for Multi-Lab SWATH Acquisition Performance Study

(note: blue font below indicates the all of the info contained in the standard operating procedure)

In order to assess system suitability of the nano LC-MS systems at the individual participating sites, all users were asked to perform two different system suitability tests prior to starting the main SWATH study. Two different sets of samples were used as QC samples, a i) predigested Beta-Galactosidase sample to assess general system performance, as well as ii) a predigested HEK293 cell line lysate to assess SWATH performance.

- Use the following buffers for the LC system (Buffer A - 2% acetonitrile in 0.1% formic acid and Buffer B - 98% acetonitrile in 0.1% formic acid).
- Recommended tips for nanoflow are the New Objectives FS 360-20-10-N-20.
- Recommended vials can be ordered from SCIEX (although if you currently use good low binding vials those are fine as well):
    - 910-00104 11 mm PP 250 µl vial (100/pkg)
    - 910-00103 11 mm snap cap with slit septum (100/pkg)

System should be set up with the cHiPLC® system in normal trap elute mode with a trap elute jumper (PN 800-00389) in the left most slot, a 75µm x 15cm column (PN 804-00001) in the middle slot, and a second 75µm x 15cm column (PN 804-00001) in the right most slot. It is recommended that new cHiPLC chips are used for this study and kept only for this study (QC samples and Dynamic range samples to follow). Set the column temperature for both column slots at 35 C.

Beta Gal Nanoflow LC System Suitability Test

First, ensure the nanoflow LC and MS system are working well using BetaGalactosidase test. This test involves a TOF MS and looped MS/MS scan to test both MS and MS/MS sensitivity. Beta-Galactosidase digestion (SCIEX, LC/MS Peptide Calibration Kit - PN 4465867) stock solutions should be made at a concentration of 1 pmol/µL (625 pmol per vial).

- Add 625.0 µL of Buffer A (100% water:0.1% formic acid) to the Beta-Galactosidase vial.

- Vortex the vial for at least 30 seconds.
- Using a centrifuge, spin the vial to bring the liquid down to the bottom of the vial before opening.
- Repeat step 2 and step 3 to confirm dissolution.
- Aliquot the stock solution (1 pmol/µL concentration) into 50 µL volumes and freeze for future use.

Dilute to 12.5 fmol/µL, such that the 2 µL injection will give 25 fmol total on column.

MS Method

Basic MS method details are shown below. The method is built into the Acquisition method folder of the Analyst® Software project to be used for this project.

**TOF MS**	Mass Range	400-1250
	Accumulation time	250 msec
	Source conditions	Optimized
	Duration	44 min
**TOF MS/MS**	Mass Range	100-1500
	Accumulation time	500 msec
	Product Ion	729.5
	MS/MS Type	High Sensitivity
	Collision Energy	42
	Collision Energy Spread	5

Pump Methods

Use the loading pump method with 500 nL/min flow rate for 30 mins for sample loading (Load 30min 0_5uLmin.ini).

Analytical gradient pump method is shown below for nanoLC 425.

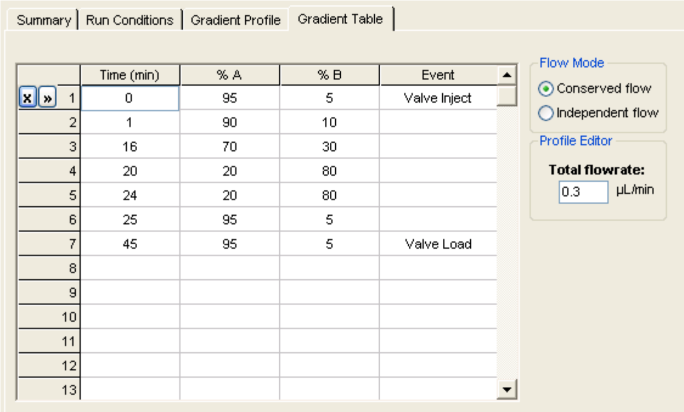

	Time (min)	% A	% B	Event
1	0	95	5	Valve Inject
2	1	90	10	
3	16	70	30	
4	20	20	80	
5	24	20	80	
6	25	95	5	
7	45	95	5	Valve Load
8				
9				
10				
11				
12				
13				

Analytical gradient pump method is shown below for nanoLC Ultra™ System.

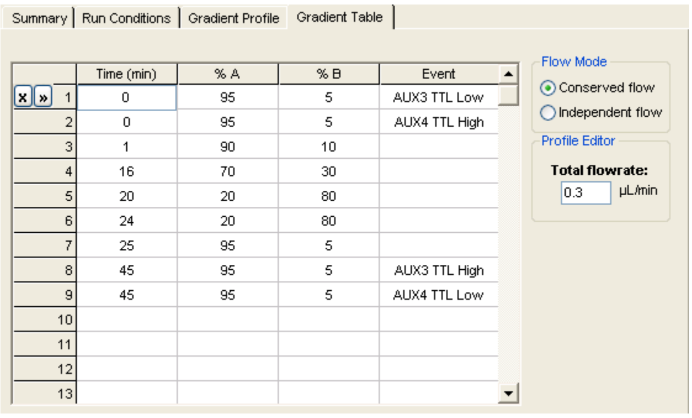

	Time (min)	% A	% B	Event
1	0	95	5	AUX3 TTL Low
2	0	95	5	AUX4 TTL High
3	1	90	10	
4	16	70	30	
5	20	20	80	
6	24	20	80	
7	25	95	5	
8	45	95	5	AUX3 TTL High
9	45	95	5	AUX4 TTL Low
10				
11				
12				
13				

All pump methods are placed in the C:\Program Files\Eksigent NanoLC\settings\method folder for use.

Set the cHiPLC System temperature for slot 2 and 3 to 35°C.

Autosampler (AS) Method

Plumb the autosampler using a 10 µL sample loop. Use an µL pick-up autosampler method of 2 µL volume.

Procedure

1. Set up the nanoflow LC and TripleTOF® 5600 system according to the instructions above.

2. Build the MS method, then add the AS and pump methods to the method to finalize and save.

3. Acquire multiple injections of the BetaGal digest with 25 fmol total on column until the system stabilizes. Once stabilized, inject a calibration method followed by three BG QC samples for replicate analysis, see file naming nomenclature below for collection of final data files.

4. Open the data files in PeakView® Software and extract the table of ions from the TOF MS data using the *Extract Ions using Dialogue* function.

Center	Width	Compound
433.87915	0.05	ELNYGPHQWR
450.69595	0.05	FNDDFSR
503.2368	0.05	YSQQQLMETSHR
528.93408	0.05	RDWENPGVTQLNR
542.2645	0.05	GDFQFNISR
550.28015	0.05	IDPNAWVER
567.0551	0.05	DVSLLHKPTTQISDFHVATR
607.8588	0.05	ITDSLAVVLQR
671.33789	0.05	VDEDQPFPAVPK
697.8694	0.05	LPSEFDLSAFLR
714.84692	0.05	DWENPGVTQLNR
729.36517	0.05	APLDNDIGVSEATR
871.9516	0.05	LSGQTIEVTSEYLFR
879.4339	0.05	VNWLGLGPQENYPDR

5. Ensure that the peak shape and widths look good, average peak width ~0.12 at half height.

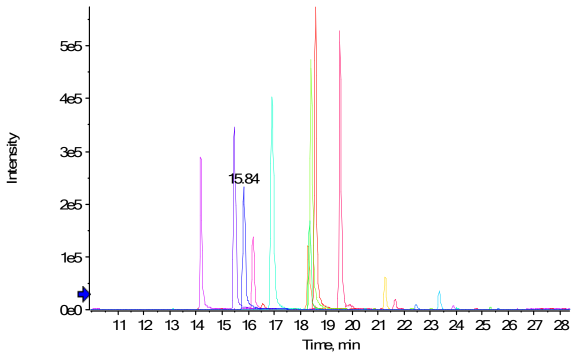

6. Ensure the peak areas for the peptides are reasonable and consistent across the 3 replicates. There will be some variation of peak areas and peaks detected depending on BG lot # but on average the peak areas should be greater than values below. Make sure the retention times

look reasonable, good separation of peaks, no big dead volume causing the peaks to come out very late, minimal peak tailing, etc.

Center	Peak Area
542.2645	300000
671.33789	600000
714.84692	150000
729.36517	600000

7. Assess the above MS1 peak area XIC's of your 3x Beta Gal acquisitions for injection to injection reproducibility.

8. From the TOF MS/MS data, extract an XIC of the 832.46 fragment ion. The peak area of the XIC should be > 7.5e4. Ensure good reproducibility of the MS/MS peak area across the three injections.

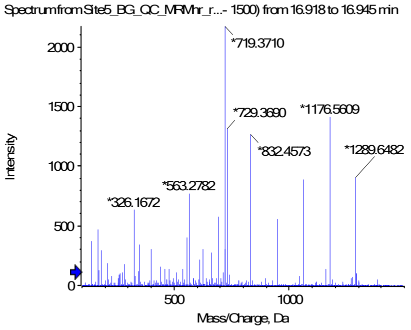

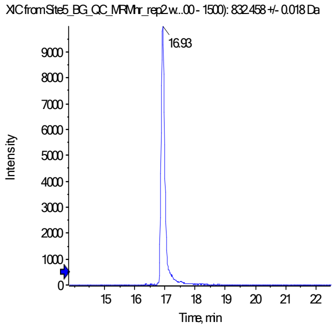

9. Ensure the mass accuracy observed on average for the TOF MS and MS/MS peaks at peak apex is better than 10 ppm. Record the information in the Excel worksheet.

10. This BetaGal QC method will be used for instrument LC QC throughout the study. It is recommended that this method is also used for instrument calibration throughout the study.

File Naming

1. Each site will be assigned a site number, in case in the future we want to share the data in an anonymous manner.

2. To facilitate organization of the data as well as processing of the data through the ETHZ analysis pipeline please use a file naming convention that includes an acronym which identifies your site, the sample injected, and the replicate number, etc).

   For example, for replicate #1 of the BG QC sample acquired at your site, the file name contains your site number and look as follows: SiteX_BGQC_MRMhr_rep1.wiff

Human HEK293 Sample SWATH-MS QC Test

After performing the BetaGal System test, the SWATH QC test can be performed.
The sample contains a digest of HEK293 cells with iRT calibration peptides added (total protein concentration is 0.5 µg/µL). Thaw the sample and then spin for 5min at high speed to remove any micro particulates before analysis. For each site a vial named "HEK293 0.5 µg/µL +iRT 1:20" with a volume of 100 µL is provided. A 2 µL injection will be made meaning the total protein on column will be 1 µg.

MS Method

To ensure consistency across the labs, please edit the IDA Collision Energy Parameters found under the Scripts menu in Analyst. The only row you need to edit is the row for the charge state 2. These are the settings used in the generation of the large Human SWATH ion library that ETH will use to process all the datasets, and these settings provide the best data when used in combination with this library. ***Note:*** This is not meant to be a recommendation of the best CE settings to use in your lab going forward, we are planning a future project to measure the optimal CE settings to use in SWATH acquisition in the future.

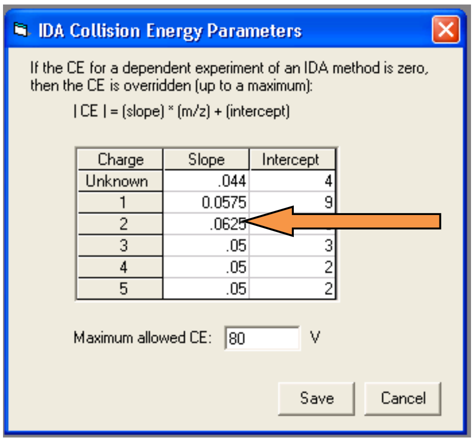

Basic method details are shown below. This method is built into the Acquisition method folder of the Analyst project to be used for this project.

**TOF MS**	Mass Range	400-1250
	Accumulation speed	250 msec
	Source conditions	Optimized
	Duration	160 min
**TOF MS/MS**	Mass Range	100-1500
	Accumulation speed	45 msec
	SWATH window – text file	Variable window file (Human HEK293 VW64_CES) 10_15.wpoa)
	MS/MS Type	High Sensitivity
	Collision Energy	Rolling CE, 2+ selected
	Cycle time	3.2 sec

This SWATH acquisition method is a variable window size method consisting of 64 windows in total with 45 msec accumulation time for each. This provides a total cycle time of 3.2 sec.
The Human HEK293 VW64_CES 10_15.wpoa will also be provided with the methods in case there are issues acquiring the supplied acquisition file. This also contains information about the variable window set-up for later processing where required.

Pump Method

Use the loading pump method with 500 nL/min flow rate for 30 mins for sample.
Analytical gradient pump method is shown below for nanoLC™ 425 system.

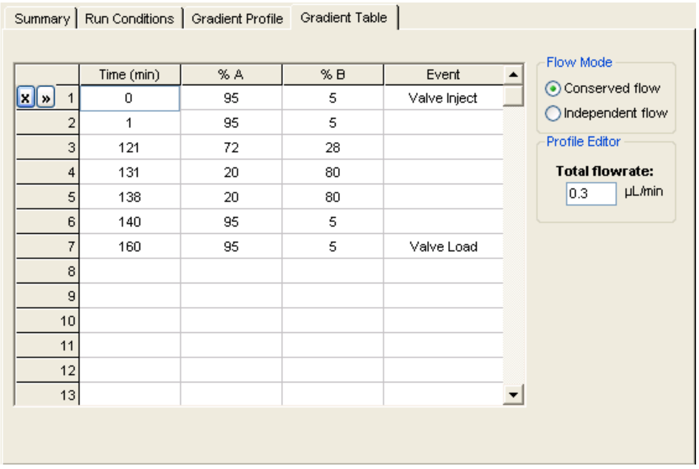

	Time (min)	% A	% B	Event
1	0	95	5	Valve Inject
2	1	95	5	
3	121	72	28	
4	131	20	80	
5	138	20	80	
6	140	95	5	
7	160	95	5	Valve Load
8				
9				
10				
11				
12				
13				

Analytical gradient pump method is shown below for nanoLC Ultra.

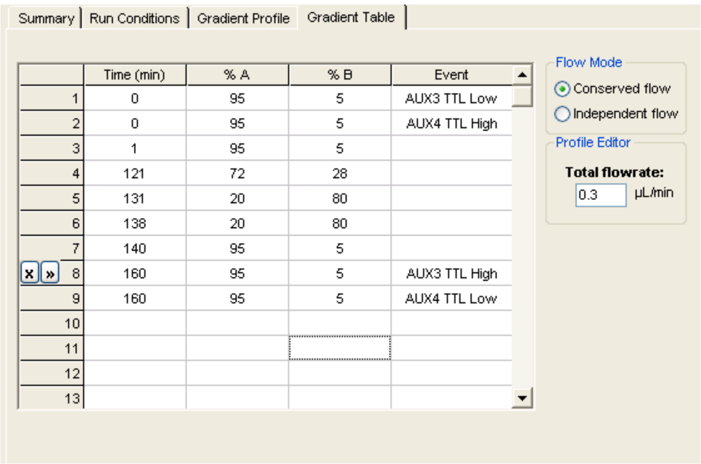

	Time (min)	% A	% B	Event
1	0	95	5	AUX3 TTL Low
2	0	95	5	AUX4 TTL High
3	1	95	5	
4	121	72	28	
5	131	20	80	
6	138	20	80	
7	140	95	5	
8	160	95	5	AUX3 TTL High
9	160	95	5	AUX4 TTL Low
10				
11				
12				
13				

All pump methods are placed in the C:\Program Files\Eksigent NanoLC\settings\method folder for use.

Autosampler Method

Plumb the autosampler using a 10 µL sample loop. Use an µL pick-up autosampler method of 2 µL volume.

Procedure

2. Set up the nanoflow LC and TripleTOF 5600 system according to the instructions above.

3. Build the MS method, then add the AS and pump methods to the method to finalize and save.

4. Ensure that the collision energy settings in the *Scripts → IDA CE Parameters* menu is set as described above.

5. Do a couple injections of the HEK lysate at the 1 µg on column load to ensure the method acquisition is fine and the separation looks good.

6. Once the system looks good, perform a calibration injection first, then acquire at least 5 replicate injections of the HEK_iRT sample with 1 µg total protein on column (2 µL injection). Ensure that good chromatographic and TIC intensity reproducibility is achieved.

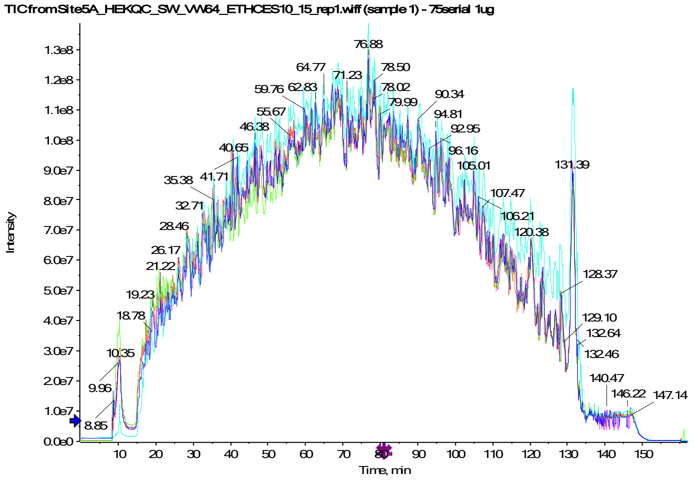

7. Extract iRT peptides across the HEK replicates using the provided iRT Ion Library file (iRT Ion library.txt) and the SWATH 2.0 software. Evaluate the peak shape and intensities of these peaks.

8. Send the data to the FTP site for central processing.

File Naming

1. Each site will be assigned a site number, in case in the future we want to share the data in an anonymous manner. Contact Christie for your site number.

2. To facilitate organization of the data as well as processing of the data through the ETHZ analysis pipeline please use a file naming convention that includes an acronym which identifies your site, the sample injected, and the replicate number, etc).

   For example, for replicate #1 of the BG QC sample acquired at your site the file name should be: Site1_HEKQC_SW_rep1.wiff

DataFile Sharing

1. Once the acquisition is complete, upload a representative BetaGal datafiles and the SWATH HEK replicates for processing and sharing.
2. These files will be analyzed centrally to measure reproducibility and test the pipeline.
3. Results will be shared with the team as they are generated.

Human HEK293 Sample SWATH Dynamic Range Experiment

Once the results for the QC samples have been evaluated, the next round of samples will be sent out for analysis.
These samples consist of the same HEK293 cell lysate with iRT peptides as a matrix but also have synthetic peptides dosed in across a broad dynamic range. There are 5 samples with different peptide dosing, so we can measure fold change between samples and dynamic range within samples. In addition, metrics on inter and intra-day reproducibility can be assessed. Each peptide group will have 6 or more different peptides and was dosed into each sample at increasingly lower amounts. The Dynamic Range samples have only enough for two attempts at the batch so please make sure everything is performing well before submitting the final batch.

Sample amounts in shipment:

Sample	Total Volume Supplied (uL)	# Injections Planned	Volume of Injection (uL)	Total Volume Required (uL)
S1	25	3	2	6
S2	25	3	2	6
S3	25	3	2	6
S4	60	9	2	18
S5	15	3	2	6

LC-MS Method

The samples will be acquired using the similar method as before but with the new analytical gradient substituted in the previous SWATH method. Two µL injections will be used as before resulting in 1µg of HEK 293 matrix on column. Please replace the gradient in the previous SWATH acquisition method with the method below and compare the shape of your TIC to that shown below. If you need to slightly modify the gradient to achieve a similar TIC as below, please do and record the gradient that you used.

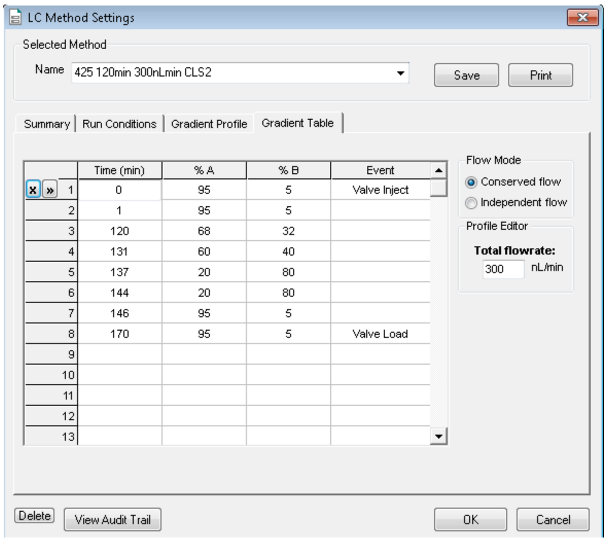

Set the cHiPLC System temperature for slot 2 and 3 to 35°C.

Procedure

1. Repeat the BGal MRM^{HR} workflow and SWATH HEK_QC test as described in Sections 1 and 2, and ensure that your system is performing as before. Example of TIC to try to obtain test using the HEK matric from the SWATH QC round, with new optimized gradient.

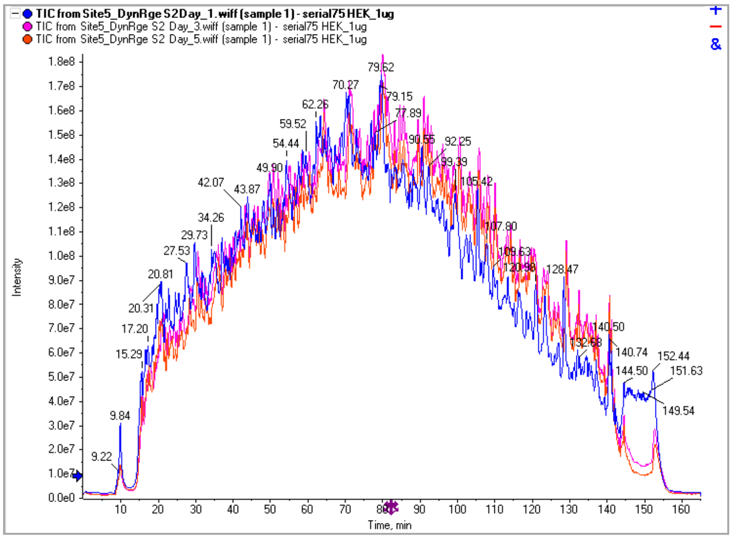

2. Once you are satisfied with your current system performance you are ready to submit the Dynamic Range Batch. If you have doubts check back with Christie.
3. Build an Analyst batch following the batch structure shown below. Have the Beta Gal calibration set to acquire after every 3 injections. The samples are acquired from low concentration (S1) to high concentrations (S5). Sample 4 is acquired in triplicate to provide the intra-day reproducibility measurements. Each sample will be run on successive days for the inter day reproducibility. For the days 2 and 4, these can be filled with BG Cal injections and the occasional HEK_QC injection to check performance.

<table>
<tr><th>Injection #</th><th>Day 1</th><th>Day 2</th><th>Day 3</th><th>Day 4</th><th>Day 5</th><th>Day 6</th></tr>
<tr><td>1</td><td>BG Cal</td><td rowspan="12">Samples Finishing - Fill day with BG runs (include HEK_QC if you want to check performance)</td><td>BG Cal</td><td rowspan="12">Samples Finishing - Fill day with BG runs (include HEK_QC if you want to check performance)</td><td>BG Cal</td><td rowspan="12">Samples Finishing</td></tr>
<tr><td>2</td><td>S1</td><td>S1</td><td>S1</td></tr>
<tr><td>3</td><td>S2</td><td>S2</td><td>S2</td></tr>
<tr><td>4</td><td>S3</td><td>S3</td><td>S3</td></tr>
<tr><td>5</td><td>BG Cal</td><td>BG Cal</td><td>BG Cal</td></tr>
<tr><td>6</td><td>S4 A</td><td>S4 A</td><td>S4 A</td></tr>
<tr><td>7</td><td>S4 B</td><td>S4 B</td><td>S4 B</td></tr>
<tr><td>8</td><td>S4 C</td><td>S4 C</td><td>S4 C</td></tr>
<tr><td>9</td><td>BG Cal</td><td>BG Cal</td><td>BG Cal</td></tr>
<tr><td>10</td><td>S5</td><td>S5</td><td>S5</td></tr>
<tr><td>11</td><td>BG</td><td>BG</td><td>BG</td></tr>
<tr><td>12</td><td>BG</td><td>BG</td><td>BG</td></tr>
</table>

4. Use the file naming as suggested above in the batch. For example, Site#_DynRange S1 Day_1 represents the first sample S1 acquisition on the first Day by a specific site. Site#_DynRange S4 Day_3B represents the second replicate of the fourth sample S4 acquisition on the 3rd day by a specific site.
5. The iRT peptides are included in the HEK matrix in the dynamic range sample so you can check your performance throughout your long batch by monitoring the sensitivity of the iRT SWATH results as above.

6. Upload the final dataset to the FTP site as described above.

References

1. Reiter, L. *et al.* Protein Identification False Discovery Rates for Very Large Proteomics Data Sets Generated by Tandem Mass Spectrometry. *Mol. Cell. Proteomics* **8,** 2405–2417 (2009).
2. Reiter, L. *et al.* mProphet: automated data processing and statistical validation for large-scale SRM experiments. *Nat. Methods* **8,** 430–435 (2011).
3. Storey, J. D. & Tibshirani, R. Statistical significance for genomewide studies. *Proc. Natl. Acad. Sci.* **100,** 9440–9445 (2003).
4. Rosenberger, G. *et al.* A repository of assays to quantify 10,000 human proteins by SWATH-MS. *Sci. Data* **1,** 140031 (2014).
5. Rosenberger, G. *et al.* Considerations for peptide and protein error-rate control in large-scale targeted DIA analyses. (submitted manuscript)
6. Röst, H. L. *et al.* OpenSWATH enables automated, targeted analysis of data-independent acquisition MS data. *Nat. Biotechnol.* **32,** 219–223 (2014).
7. Schubert, O. T. *et al.* Absolute Proteome Composition and Dynamics during Dormancy and Resuscitation of Mycobacterium tuberculosis. *Cell Host Microbe* **18,** 96–108 (2015).
8. Navarro, P. *et al.* A multicenter study benchmarks software tools for label-free proteome quantification. *Nat. Biotechnol.* **34,** 1130–1136 (2016).
9. Röst, H. L. *et al.* TRIC: an automated alignment strategy for reproducible protein quantification in targeted proteomics. *Nat. Methods* **13,** 777–783 (2016).
10. Karpievitch, Y. V., Dabney, A. R. & Smith, R. D. Normalization and missing value imputation for label-free LC-MS analysis. *BMC Bioinformatics* **13,** S5 (2012).
11. Escher, C. *et al.* Using iRT, a normalized retention time for more targeted measurement of peptides. *Proteomics* **12,** 1111–21 (2012).
12. Schubert, O. T. *et al.* Building high-quality assay libraries for targeted analysis of SWATH MS data. *Nat. Protoc.* **10,** 426–441 (2015).

13. Tabb, D. L. *et al.* Repeatability and Reproducibility in Proteomic Identifications by Liquid Chromatography−Tandem Mass Spectrometry. *J. Proteome Res.* **9,** 761–776 (2010).
